# Oral contraceptive usage among healthcare workers and its impact on COVID-19 booster vaccination immunogenicity

**DOI:** 10.1038/s41541-026-01510-z

**Published:** 2026-07-02

**Authors:** Isabell Wagenhäuser, Julia Reusch, Juliane Mees, Lukas B. Krone, Isabella Eiter, Thiên-Trí Lâm, Alexandra Schubert-Unkmeir, Carolin Curtaz, Anna Frey, Oliver Kurzai, Stefan Frantz, Sabine Wicker, Achim Wöckel, Nils Petri, Alexander Gabel, Manuel Krone

**Affiliations:** 1 Central Laboratory Unit, University Hospital Würzburg, Würzburg, Germany; 2 Department of General, Visceral, Transplantation, Vascular, and Paediatric Surgery, University Hospital Würzburg, Würzburg, Germany; 3 Centre for Neural Circuits and Behaviour, University of Oxford, Oxford, UK; 4 University Hospital of Psychiatry and Psychotherapy, University of Bern, Bern, Switzerland; 5 Institute for Hygiene and Microbiology, Julius-Maximilians-Universität Würzburg, Würzburg, Germany; 6 Department of Obstetrics and Gynaecology, University Hospital Würzburg, Würzburg, Germany; 7 Department of Internal Medicine I, University Hospital Würzburg, Würzburg, Germany; 8 Leibniz Institute for Natural Product Research and Infection Biology – Hans-Knoell-Institute, Jena, Germany; 9 Occupational Health Service, University Hospital Frankfurt, Frankfurt am Main, Germany; 10 Department of Molecular Virology, Heidelberg University, Heidelberg, Germany

**Keywords:** Diseases, Health care, Immunology, Medical research, Microbiology

## Abstract

Oral contraceptives (OCs) can substantially modulate immune responses; however, their impact on vaccine immunogenicity remains poorly understood. This study investigated OC use patterns and their determinants among healthcare workers (HCWs), and the effects of OCs on humoral and T-cell-mediated immune responses following COVID-19 booster vaccination. From 29 September 2021 to 31 December 2023, 1061 female HCWs aged 18-50 years were enrolled in the CoVacSer study. OC users were compared to non-users. Blood samples combined with a questionnaire were collected before and after third and fourth COVID-19 vaccination including follow-ups. Anti-SARS-CoV-2-Spike IgG levels were measured using SERION ELISA *agile* SARS-CoV-2 IgG, T-cellular immune response using Oxford Immunotec T-SPOT®.COVID. A linear mixed and a generalised linear model were used to assess the influence of OC on post-vaccination immune response. At study inclusion, 21.7% (230/1061) reported OC use. Younger age, being a physician, and higher BMI were significantly associated with OC. Linear mixed and generalised linear regression models revealed no significant association between reported OC use and humoral and T-cellular immune response before and after COVID-19 vaccination. Although OC are known to modulate immune responses, this study found no statistically significant association between self-reported OC use and humoral or cellular immunogenicity following COVID-19 vaccination. This is among the first studies to address this in the context of COVID-19 booster vaccination. Our findings suggest that in women using OC no specific adjustments to COVID-19 vaccination strategies are required considering OC use. Continued investigation of potential interactions between OC and vaccine- or infection-induced immunity remain warranted.

## Introduction

Oral contraceptives (OC) stand as a prevalent, long-term medication widely utilized among the female population. Oral contraceptives include both pure progestins and oestrogen/progestin combination preparations^1,2^. The United Nations contraception use report quantifies the mean OC prevalence rate for contraception among women in the reproductive age with 8% globally and 19% in Europe^3^. In Germany, statistics indicate that 32% of women aged 18 to 21 years were prescribed OC in 2021, covered by statutory health insurance^4^. This represents a notable decline from peak levels up to 46% in 2010. Nevertheless, the use of OC in this demographic remains substantial^5^. Further survey-based data from the German Federal Centre for Health Education (BZgA) reveals that in 2023, 26% of women aged 18 to 49 reported utilising OC for contraception, indicating a decreasing trend from 2011, where the rate was 40%^6,7^.

Among healthcare workers (HCWs), a population that includes a substantial proportion of women of reproductive age, existing evidence examines factors influencing OC uptake. These factors include the potential to control one’s own menstrual cycle by OC, environmental influences, specific occupational roles within the healthcare sector, and the notable consideration given during professional training^8–10^.

The relationship between OC use and the immune system, particularly the potential impact of prolonged OC use on the immunogenicity of vaccinations and infections, remains inadequately explored^11^. Existing evidence suggests a potential modulation of the immune system by OC use with regard to a possible acceleration of the occurrence of certain autoimmune diseases^12^, a significantly increased risk of certain infections (bronchopneumonia, cellulitis, acute and streptococcal pharyngitis, aphthous stomatitis) under OC use^13^, as well as the influence on cytotoxic lymphocytes and cytokines throughout the menstrual cycle^14–16^. Other studies, however, report no differences in the proliferative immune response of humoral or T-cells in women using OC compared to controls^17^.

Despite these studies, it remains largely unknown whether OC affects the immunogenicity of vaccinations in general, and particularly COVID-19 vaccinations^18^. Amidst this exploration, HCWs emerge as a pivotal study group, representing a highly exposed cohort against infectious diseases. Examining the complex relationship between OC and immune responses in this population not only sheds light on potential effects on individual health, but also has broader implications for public health strategies, including infection prevention and control. These considerations underscore the need for more nuanced research into the interplay between medical interventions and immune system resilience.

This study is the first to investigate OC use behaviour and its influencing factors in HCWs and the effects of OC on the humoral and T-cellular immune response after COVID-19 encompassing booster vaccinations.

## Methods

This study was conducted as part of the CoVacSer study, a prospective longitudinal study investigating COVID-19 vaccination and SARS-CoV-2 infection related immunity, its influencing factors, and the relation to quality of life and ability to work in HCWs lasting from 29 September 2021 to 31 December 2023.

The general inclusion criteria according to the CoVacSer study protocol were as following^19^:

- age ≥ 18 years
- written informed consent
- minimum interval of 14 days after the first PCR-confirmed SARS-CoV-2 infection and/or at least one dose of European Medicines Agency (EMA) authorised COVID-19 vaccine^20^
- COVID-19 vaccination regardless of vaccination schedule at study inclusion
- employment in the healthcare sector

Individuals vaccinated with at least one dose of a non-EMA-authorised COVID-19 vaccine were excluded^20^.

A serum blood sample was collected for each study participant in combination with the CoVacSer study questionnaire at each participation. Only blood samples with a signed written consent form and completed linked questionnaire were considered. REDCap (Research Electronic Data Capture, projectredcap.org) was used for questionnaire recording^21,22^.

The CoVacSer study survey includes socio-demographic aspects and individual risk factors, containing the World Health Organization Quality of Life (WHOQOL-BREF) questionnaire and the Work Ability Index^23–25^. As part of this survey, the OC use was assessed. The selection option in the questionnaire for long-term medication was “Do you take medication regularly (multiple choice)?” with “hormonal contraception” as an option. For data protection reasons, the exact preparation, including the dosage regimen and the individual OC indication, was not requested.

The majority of HCWs were enlisted primarily from a single tertiary hospital in Germany, supplemented by the inclusion of some HCWs from nearby healthcare facilities.

The following inclusion criteria were defined in addition to the general inclusion criteria:

- age between 18 and 50 years (inclusive)
- female gender
- self-reporting of OC use

Only individuals fulfilling general and additional criteria and participating within the intervals defined in the study protocol were included.

The analysis was conducted for each subordinate issue in the following two cohorts: ‘OC’ (continuous OC medication) and ‘no OC’ (no continuous OC medication).

Two cross-sectional time points during the entire study period were analysed: study inclusion (first study participation 29 September to 12 November 2021) and second cross-section of the overall study cohort regardless of the follow-up indications (1 September to 30 September 2023).

The evaluation of the influence of OC use on SARS-CoV-2 related immunity was investigated in comparison with the control group without OC use at the following COVID-19 immunising events: third dose (‘first booster’) and fourth dose of COVID-19 vaccine administration (‘second booster’).

For each COVID-19 immunisation event, the following study time points were used to assess the influence of OC:

- baseline participation (last participation before vaccination, ‘pre’)
- post-vaccination participation (14-day follow-up, ‘post’)
- if available (non-mandatory inclusion criterion): participations 3 and 6 months after vaccination without further vaccination or infection during this period were considered

The assessment at the further follow-up times (12 months, 24 months, additional follow-up after seasonal Influenza vaccination without simultaneous COVID-19 vaccination)^26^ defined in the study protocol was omitted due to very few / no data points without further vaccination / infection in the meantime.

Only study participants meeting the following criteria were included in these two analyses:

- baseline participation maximum of 100 days before the third or 200 days before the fourth vaccination
- first participation post-vaccination between 14 and 59 days after vaccination
- complete indication of individual COVID-19 vaccination data
- possible Anti-SARS-CoV-2-Spike IgG determination

The analysis of the influence of long-term OC use on COVID-19-related immunity was supplemented by the analysis of cellular immunity. The T-SPOT®.COVID test was used to quantify the T-cellular reactivity, whereby these data was obtained in the period from 2 March 2022 to 9 May 2022, in which heparin blood samples could be voluntarily sent in addition to the serum samples at regular study participation.

Only samples meeting the following criteria were included for this subanalysis:

- valid T-SPOT®.COVID result
- parallel submission of serum and heparin blood sample (maximum 14 days interval in-between)
- complete indication of individual COVID-19 vaccination data
- possible parallel Anti-SARS-CoV-2-Spike IgG determination
- threefold COVID-19 vaccination

In case of multiple T-SPOT®.COVID test results, the chronologically first one was included.

Anti-SARS-CoV-2-Spike IgG levels in Binding Antibody Units per ml (BAU/ml) were assessed using the SERION ELISA *agile* SARS-CoV-2 IgG (SERION diagnostics, Würzburg, Germany), an enzyme-linked immunoassay which has proved a high agreement with a neutralising assay^27^.

T-cellular reactivity against SARS- CoV-2 Spike S1 and Nucleocapsid antigen was assessed using the T-SPOT®.COVID test (Oxford Immunotec Ltd., Abingdon, UK) as ELISpot (enzyme-linked immunospot) IGRA (interferon-gamma (IFN-γ) release assay)^28^. Previous studies have demonstrated the T-SPOT®.COVID test’s performance and reliability in detecting SARS-CoV-2 sensitised T-cells^29–32^.

The obtained spot counts as T-cellular reactivity measure were obtained as spot-forming units (SFU; spots per 1×10^6^ peripheral blood mononuclear cells (PBMCs)).

The study protocol was approved by the Ethics Committee of the University of Würzburg in accordance with the Declaration of Helsinki (file no. 79/21). The CoVacSer study is registered at the Paul Ehrlich Institute (PEI, German Federal Institute for Vaccines and Biomedicines; non-interventional study (NIS) No. 674).

Data analysis was performed using GraphPad Prism 11.0.0 (GraphPad Software, San Diego, CA, USA) and the statistical programming language R (version 4.3.1).

For data protection reasons, as the number of cases was too small, subgroups were partially summarised when visualising age categories and household size.

For characterisation of the two cohorts (OC vs. no OC) in total and in the sub-cohorts the following statistical tests were used:

The Fisher’s exact test was used for the binary variables smoking, COVID-19 vaccine, SARS-CoV-2 infection, congenital or acquired immunodeficiency. The Chi square test was used for the comparison of COVID-19 vaccine, SARS-CoV-2 infection, occupational groups (categories: nursing, medical service, other activity with regular patient contact, other activity without regular patient contact), frequency of patient contact (categories: never, rare, regularly, often, very often), long-term medication (categories: analgesics, systemic immunosuppressive, or immunomodulation drugs, glucocorticoids, other). The Mann Whitney U test was used for comparing the ordinal variables age, BMI, number of household members, interval from the pre- and post-event participation to the respective referring dose of COVID-19 vaccination or SARS-CoV-2 infection, interval since last COVID-19 immunising event, Anti-SARS-CoV-2-Spike and Anti-SARS-CoV-2-Nucleocapsid SFU levels.

Confidence intervals were calculated using the Wilcoxon-Brown Method^33^.

A logistic regression analysis was performed to investigate the association between OC use and characteristic factors within the study population such as age, occupational groups (reference: nursing), BMI, household size and smoking.

Evaluating the statistical differences between the two cohorts OC and no OC concerning Anti-SARS-CoV-2-Spike IgG levels post third and fourth COVID-19 vaccination, a linear mixed model was conducted. It was assumed that IgG levels follow a log-normal distribution. For the third COVID-19 vaccination the time points pre-vaccination, post-vaccination, and 3-month post-vaccination were included in the model. Adding the 6-month post vaccination time point reduced the size of the study population from 718 to 198 due to missing follow-up data. Hence, to use the dataset in its extent and investigate statistical differences after 6 months, a disjunct model was conducted including all four time points. A separate model was also used to analyse the differences OC and no OC after the fourth COVID-19 vaccination.

Determining the statistical differences between OC and no OC in terms of T-cell activation, a generalised linear model was used. It was assumed that the observed spot counts for Anti-SARS-CoV-2-Spike and Anti-SARS-CoV-2-Nucleocapsid SFU follow a negative binomial distribution. Both models incorporated several covariates, including smoking habits, occupational group, history of SARS-CoV-2 infection, vaccine type, age, number of household members, and body mass index (BMI), to account for potential confounding factors. Subsequently, the statistical differences between OC and no OC cohorts regarding IgG levels or T-cell activation were estimated using marginal means derived from the established models.

Moreover, the statistical differences among covariate groups were determined by the same approach.

To correct against multiple testing, the resulting *p*-values were adjusted using the Benjamini-Yekutieli procedure^34^. Adjusted *p*-values < 0.05 were considered statistically significant.

## Results

**Fig. 1:**
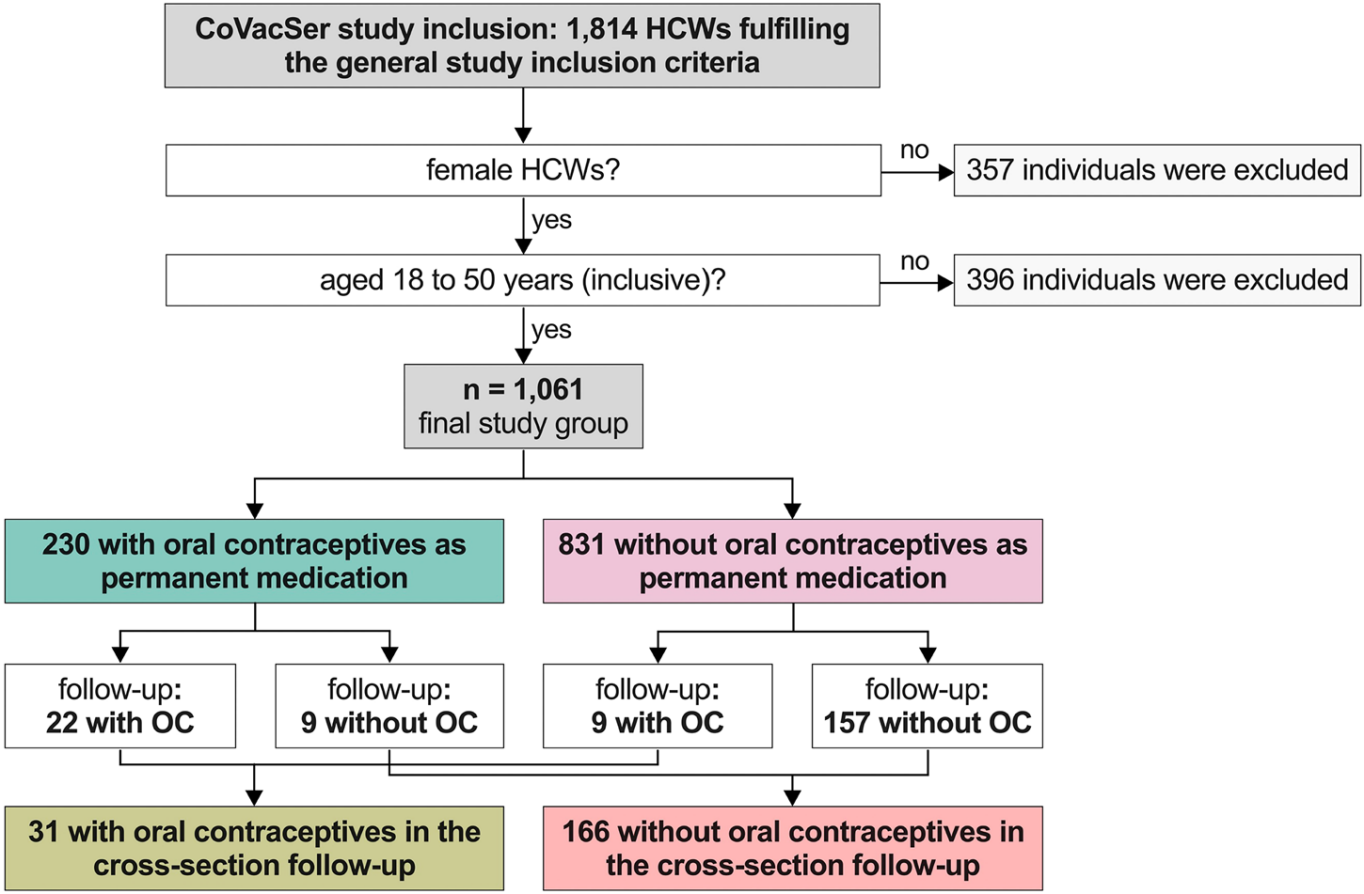
Subject inclusion for the analysis of the overall OC use among HCWs including follow-up in the second cross-sectional phase. follow-up: cross-section follow-up in September 2023. OC: oral contraceptives.

**Fig. 2:**
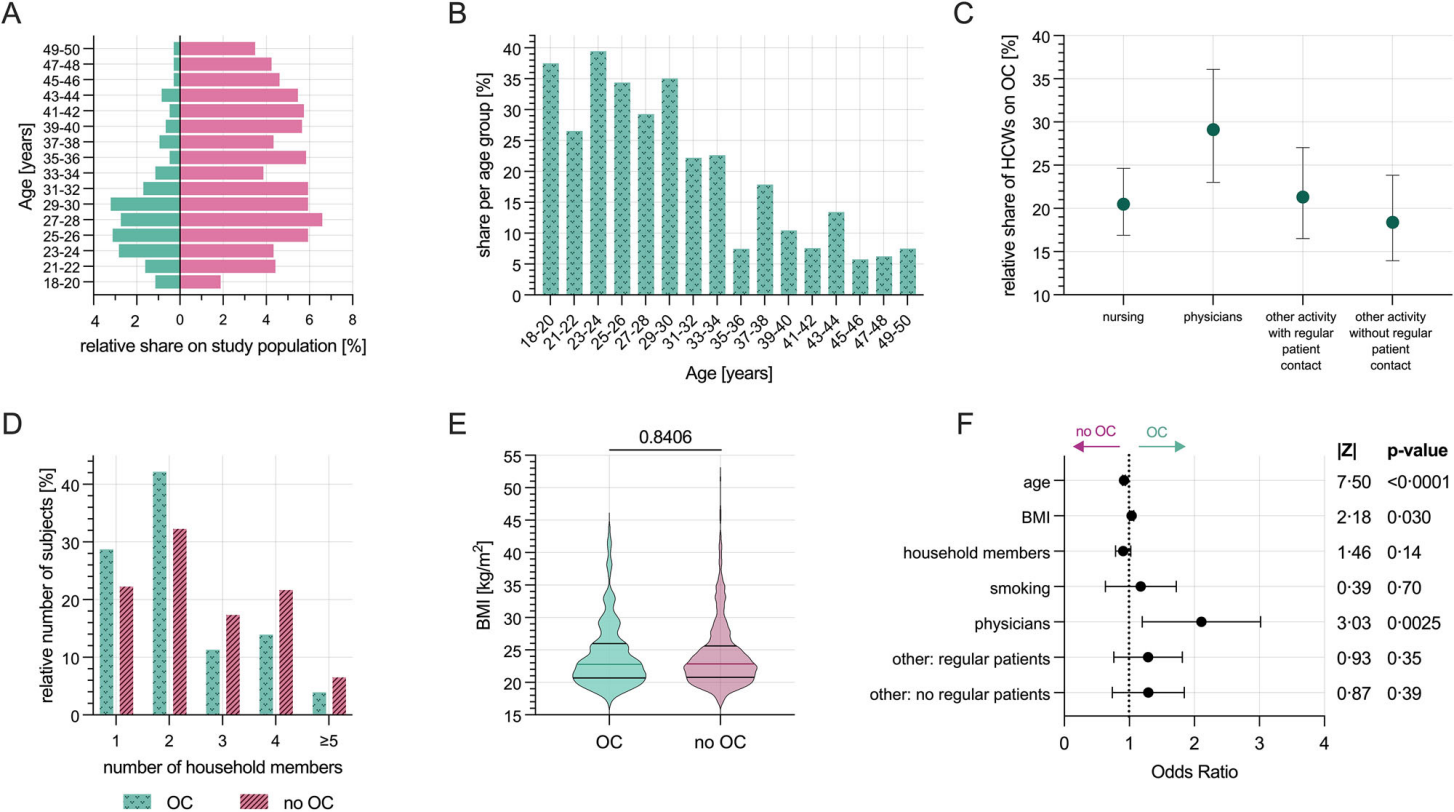
Characterisation of the study population at study inclusion stratified by OC use (individuals with OC use: *n* = 230 (21.7%), individuals without OC use: *n* = 831 (78.3%)). **A** Age structure stratified by OC use. **B** Relative share of HCWs per age group with OC use. **C** Relative share of subjects with OC use stratified by profession. **D** Relative share of subjects stratified by number of household members and OC use. **E** BMI stratified by OC use. **F** Odds Ratio of potential factors influencing OC use. In the case of whiskers in the figures, these represent the respective 95% confidence interval. BMI: Body Mass Index [kg/m^2^]. OC: oral contraceptives.

At study inclusion, 1061 female HCWs aged 18 to 50 years met all inclusion criteria regarding participation indication and time according to the study protocol. 230 (21.7%; 95% CI 19.3-24.3%) HCWs reported OC use, while 831 HCWs did not. The detailed characteristics of this cohort are described in Fig. 1, Table 1, and Fig. 2.

**Table 1:** Characterisation of the study population for overall usage of OC among HCWs at study inclusion

	total	OC	no OC	p	p adj.
number of subjects	1061 (100.0%)	230 (21.7%)	831 (78.3%)		
age (IQR) [years]	32 (26-41)	28 (24-32)	35 (27-42)	<0.0001	0.0011
BMI (IQR) [kg/m^2^]	22.7 (20.8-25.7)	22.8 (20.7-26.0)	22.9 (20.8-25.6)	0.84	1.00
household size (IQR)	2 (2-4)	2 (1-3)	2 (2-4)	<0.0001	0.0011
*smoking*					
smoking	135 (12.7%)	29 (12.6%)	106 (12.8%)	1.00	1.00
non-smoking	926 (87.3%)	201 (87.4%)	725 (87.2%)		
*profession*					
nursing	415 (39.1%)	85 (37.0%)	330 (39.7%)	0.90	1.00
physicians	182 (17.2%)	53 (23.0%)	129 (15.5%)		
other activity with regular patient contact	230 (21.7%)	49 (21.3%)	181 (21.8%)		
other activity without regular patient contact	234 (22.1%)	43 (18.7%)	191 (23.0%)		
frequency of patient contact					
never	150 (14.1%)	29 (12.6%)	121 (14.6%)	0.26	1.00
rare	103 (9.7%)	20 (8.7%)	83 (10.0%)		
regularly	122 (11.5%)	19 (8.3%)	103 (12.4%)		
often	97 (9.1%)	21 (9.1%)	76 (9.1%)		
very often	589 (55.5%)	141 (6 1.3%)	448 (53.9%)		
*congenital or acquired immunodeficiency*					
immunodeficiency	27 (2.5%)	7 (3.0%)	20 (2.4%)	0.64	1.00
no immunodeficiency	1,034 (97.5%)	223 (97.0%)	811 (97.6%)		
*long-term medication*					
analgesics (e.g. ibuprofen, metamizole/novaminesulfone)	50 (4.7%)	13 (5.7%)	37 (4.5%)	0.64	1.00
systemic immunosuppressive or modulating drugs (e.g. cortisone, antibody therapies)	20 (1.9%)	4 (1.7%)	16 (1.9%)		
inhalative or topical glucocorticoids (e.g. budesonide)	27 (2.5%)	2 (0.9%)	25 (3.0%)		
other	241 (22.7%)	50 (21.7%)	191 (23.0%)		

The relative numbers in relation to the number of subjects of the study population are given in brackets following the absolute number. Age, BMI, and household size are given as medians with interquartile ranges in parentheses.

Where absolute figures are given, the corresponding relative figures are given in brackets. These always refer to the number of individuals in the respective categorical column. The exception is the relative numbers in relation to the column category, which refer to the total study population.

BMI: Body Mass Index [kg/m^2^].

OC: oral contraceptives.

The HCWs with OC use were significantly younger (*p* < 0.0001; p-adj.=0.0011) and lived in significantly smaller households (*p* < 0.0001; p-adj.=0.0011). There were no significant differences between the two cohorts regarding BMI, smoking, occupational group, patient contact, immunodeficiency, and use of various other long-term medications (Supplementary Table 1, Fig. 2).

In the logistic regression analysis, younger age (*p* < 0.0001), being a physician (*p* = 0.0025), and higher BMI (*p* = 0.030) were found to be significantly associated with OC use. However, the factors household size (*p* = 0.14), smoking (*p* = 0.70), and the occupational categories of other HCWs with (*p* = 0.35) and without (*p* = 0.39) regular patient contact did not show a significant association (Fig. 2F).

The corresponding analysis of OC use among HCWs at the cross-sectional follow-up can be found in the *Supplementary Results*, in Supplementary Table 1, and Supplementary Figure 1.

Out of the 1061 female HCWs, 795 individuals participated according to the study protocol after their third COVID-19 vaccination with available baseline participation as reference. Considering the inclusion criteria, 718 HCWs were included (Supplementary Fig. 2), whereby 422 (58.8%) of these HCWs also participated in the 3-month follow-up and 198 (27.6%) in the 6-month follow-up without a new COVID-19 immunising event in the meantime (neither further vaccination nor further infection).

Out of the third-vaccination study cohort, 140 HCWs (19.6%) indicated OC at the time of vaccination, 578 (80.4%) of the HCWs did not. HCWs with OC had a significantly higher share of individuals aged < 30 years (*p* < 0.0001; p-adj.=0.0027) and thus were significantly less likely to receive mRNA-1273 as a third COVID-19 vaccine dose (*p* = 0.0006; p-adj.=0.0044). All characteristics of the total cohort for the analysis of the third COVID-19 vaccination as a whole and stratified by OC use are listed in Supplementary Table 2 and Supplementary Fig. 3.

**Fig. 3:**
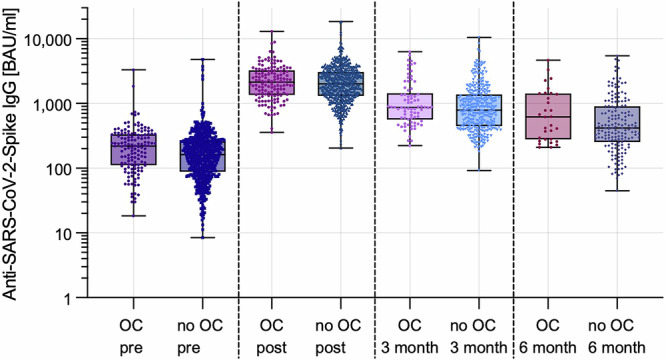
Anti-SARS-CoV-2-Spike IgG levels related to the third COVID-19 vaccination stratified by OC (individuals with OC use: *n* = 140 (19.5%), individuals without OC use: *n* = 578 (80.5%)). BAU/ml: Binding Antibody Units per millilitre. OC: oral contraceptives.

Regarding the Anti-SARS-CoV-2-Spike IgG levels before and after the third COVID-19 vaccination, the linear mixed model analysis performed with the respective target variable Anti-SARS-CoV-2-Spike IgG levels could not reveal any statistically significant differences of IgG levels with respect to OC use (p = 0.078 (p-adj.=0.32) and p = 0.41 (p-adj.=1.00) including the 6-month time point, Fig. 3 and Supplementary Statistical Analysis).

Out of the 1061 female HCWs, 115 individuals participated according to the study protocol after their fourth COVID-19 vaccination with an available baseline participation. Considering the defined inclusion criteria 72 HCWs were included (Supplementary Fig. 4), whereby 49 (68.1%) of these HCWs also participated in the 3-month follow-up and 30 (41.7%) in the 6-month follow-up without a new COVID-19 immunisation in the meantime.

**Fig. 4:**
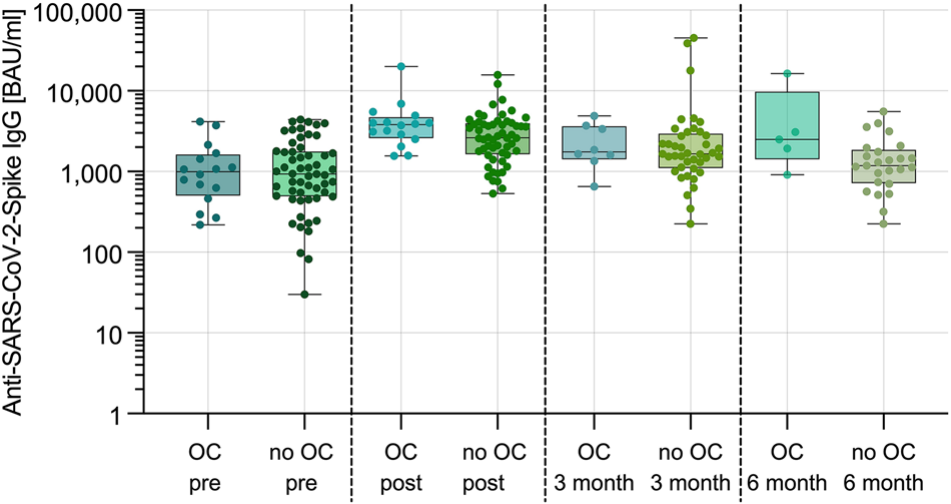
Anti-SARS-CoV-2-Spike IgG levels related to the fourth COVID-19 vaccination stratified by OC. BAU/ml Binding Antibody Units per millilitre, OC oral contraceptives, pre baseline participation (last participation before vaccination, post post-vaccination participation (14-day follow-up), 3 month 3-month follow-up after vaccination, 6 month 6-month follow-up after vaccination.

Out of the fourth-vaccination study cohort, 16 HCWs (22.2%) indicated OC use, 56 (77.8%) of the HCWs did not. All further characteristics of the total cohort for the analysis of the fourth COVID-19 vaccination as a whole and stratified by OC use are listed in Supplementary Table 3 and Fig. 4.

Regarding the Anti-SARS-CoV-2-Spike IgG levels before and after the fourth COVID-19 vaccination, the linear mixed model analysis performed with the respective target variable Anti-SARS-CoV-2-Spike IgG levels could not reveal any statistically significant differences of IgG levels with respect to OC use (*p* = 0.35; p-adj.=1.00; Supplementary Statistical Analysis).

Out of the 1061 female HCWs included, 201 HCWs had an available and valid T-SPOT®.COVID result and were exactly threefold COVID-19 vaccinated. Out of this cohort, 34 HCWs (16.9%; 95% CI 12.4%-22.7%) indicated OC use at the time of vaccination, 167 of the HCWs did not (Supplementary Fig. 6, Supplementary Table 4, Supplementary Fig. 7).

**Fig. 5:**
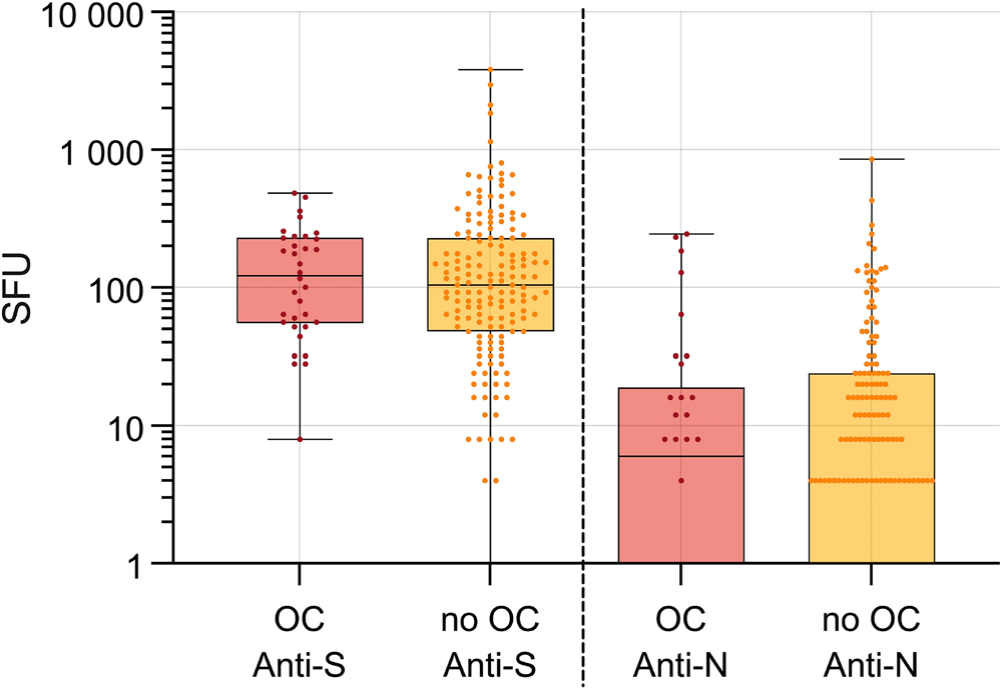
Anti-SARS-CoV-2-Spike and Anti-SARS-CoV-2-Nucleocapsid SFU among threefold vaccinated HCWs separated by OC (individuals with OC use: *n* = 34 (16.9%), individuals without OC use: *n* = 179 (89.1%)). SFU: Spot Forming Units. OC: oral contraceptives.

No significant effect of OC on Anti-SARS-CoV-2-Spike (*p* = 0.087; p-adj.=0.51) and Anti-SARS-CoV-2-Nucleocapsid (*p* = 0.84; p-adj.=1.00) SFU was obtained (Supplementary Statistical Analysis, Fig. 5).

## Discussion

To conclude, in the cohort presented of 1061 HCWs, about one fifth reported intake of OC. Consequently, the study cohort matches the general population. Younger age, working as a physician, and higher BMI correlated significantly with OC use. Linear regression mixed and generalised models revealed no significant association of OC with humoral and T-cellular immune response before and after COVID-19 vaccination including follow-ups.

The proportion of female HCWs aged 18 to 50 years who regularly use OC was 22%, which is in line with national survey-based data for the German population^6^. Looking at the sub-cohort of 18 to 21 years old women, for which precise billing data is available, 34% of the study cohort used OC. This is comparable to the 32% with OC according to the official data^4–7^.

OC was the most frequently taken medication among female HCWs. OC use was significantly more widespread among younger HCWs in smaller households, which suggests that childless or living alone HCWs might use OC for contraception reasons. Female physicians showed significantly higher rates of OC use compared to other occupational groups. This might be due to the fact that female physicians delay childbearing compared to other occupational groups. In a large long-term cohort study in Canada, Cusimano et al. were able to show that although female physicians had children just as often as the non-physician control cohort, children were born at an older age^35^. The delay of childbirth and the more frequent OC use among the female physicians might be explained by multiple factors: With self-prescription physicians have a low-threshold access to prescription-only OC in Germany. Due to a highly competitive occupational environment there is a high desire for safe contraception and the option of targeted timing and reduction of the intensity of menstruation is often a pro argument for OC use^8,35^. It is important to consider the tertiary care hospital and academic study setting, where working conditions for those pursuing scientific qualification goals entail complex clinical work and impose special demands, attracting physicians who are more oriented towards their careers. This clientele may potentially be even more inclined to resort to OC for the reasons mentioned above^35^.

Despite numerous reports of hormonal effects on single function of the immune systems^14–16,36^, in the data presented self-reported OC use did not significantly influence the humoral and cellular response to COVID-19 vaccination. This is in line with previous evidence reporting no differences in the in vitro proliferative immune response of B- or T-cells in women using OC^17^. This was demonstrated based on linear and generalised regression models for the Anti-SARS-CoV-2-Spike related humoral immune response after the third and fourth COVID-19 vaccination as well as for the effect of OC use on T-cellular Anti-SARS-CoV-2-Spike and -Nucleocapsid response after the third vaccination.

Compared to the sparse data on OC use in Germany the presented study characterises OC use in Germany for all age groups and provides insights into OC use among HCWs and how the users are characterised^4–7^. It was possible to conclusively help to address the question regarding the interaction between OC and COVID-19 vaccinations immunologically, especially since randomised controlled trials with OC in a setting of healthy individuals are not ethically justifiable. Unlike previous studies that identified correlations between OC use and single functions of the immune system by comparing OC users to control groups, but did not adequately account for confounding factors, this analysis employed a mixed-effects model to effectively control for these confounders in a best possible way^14,15^. Based on the applied statistical modelling, no evidence for an effect of OC use - a widely used long-term medication - on vaccine-induced SARS-CoV-2 immunity was observed. This study might also serve as a key pilot study for examining the immune response to further vaccinations in the context of OC use.

The study has several limitations: The study population corresponds to a cross-section of the various professional groups in the German healthcare system, particularly a German university hospital. With a broad age distribution and different occupational groups, one can assume good transferability to the overall population, but should nevertheless bear in mind the characteristics of the collective: medical staff have easier access to OC prescription and may weigh up taking them more critically if they have sound knowledge. For data protection reasons, it was not further specified which OC type - for example a pure progestin, or a combination with oestrogen - was taken, as well as which exact preparation in which dosage or which intake rhythm (intake with cyclical breaks or continuously). Consequently, potential differential effects of combined oestrogen-progestin preparations versus progestin-only contraceptives could not be assessed. Likewise, information regarding menstrual cycle phase at vaccination and blood sampling was unavailable. Therefore, formulation-specific or cycle-dependent immunological effects cannot be excluded by the present study. Futher, the use of other hormonal contraceptives like transdermal, subcutaneous, intramuscular, intrauterine, or transvaginal applications were not included. The data collected was based on a questionnaire and not on prescription statistics. However, with electronic questionnaires and standardised wording, the best possible data quality was aimed for. Only Anti-SARS-CoV-2-Spike IgG levels were measured without parallel Anti-SARS-CoV-2-Nucleocapsid IgG levels. Unknown SARS-CoV-2 infections can therefore not be ruled out. No clinical data on individual SARS-CoV-2 infection course were available. The T-cellular Anti-SARS-CoV-2 immunity was not further differentiated for different lymphocyte subpopulations, cytokine panels, or natural killer cell activity. The data do not allow conclusions regarding the quality of the immune response or direct correlates of protective immunity. Future studies should incorporate neutralisation assays, memory B cell analyses, and more detailed T cell profiling to provide a more comprehensive understanding of the immunological effects of OC use. Especially in the analysis of the fourth vaccination, subgroup sizes were relatively small, which must be considered when interpreting the data. This is mainly since especially for the follow-up time points after the fourth vaccination, with high infection rates from the Omicron VOC, only few participants reached these time points without infection between^37,38^.

To conclude, approximately one in five HCWs used OC for contraception, particularly young female physicians. In this cohort, self-reported OC use was not associated with significant differences in humoral or T-cellular immunogenicity following COVID-19 vaccination. However, because information on contraceptive formulation, hormonal composition, and menstrual cycle timing was unavailable, formulation-specific or cycle-dependent effects cannot be excluded.

This finding is relevant beyond COVID-19, as a substantial proportion of young women use OC, and may extend to future vaccines based on similar platforms, such as mRNA-based Influenza vaccines. Future research should address OC formulation-specific effects, expand cohort sizes for in-depth immunological analyses, and include more detailed immune profiling. Broader validation across diverse populations, vaccine types, and contraceptive methods, as well as longitudinal outcome data, will be important to confirm and generalise these findings.

## Supplementary information

Supplementary information

Supplementary statistical analysis

## Data Availability

Additional de-identified data underlying the results reported in this article including text, tables, figures, appendices, study protocol, statistical analysis plan, and analytic code well be made available upon reasonable request to the corresponding author for researchers providing a methodologically sound proposal to achieve the aims of the approved proposal.
