# Supplementary material for: Oral contraceptive usage among healthcare workers and its impact on COVID-19 booster vaccination immunogenicity
Source: NPJ Vaccines. 2026 Jul 2;11:134. doi: 10.1038/s41541-026-01510-z (PMC13328437; doi:10.1038/s41541-026-01510-z)
Supplement: Supplementary file 1 — Supplementary information [file 41541_2026_1510_MOESM1_ESM.pdf]

Supplementary Material

Supplementary Results

Usage of OC among HCWs at the second cross-sectional time point

A total of 418 subjects of the entire CoVacSer study collective independent of gender and age took part in the second cross-sectional survey in September 2023, 197 (47.1%) of whom were female and between 18 and 50 years old and therewith part of the initial overall cohort, forming the cohort of the cross-sectional follow-up. From this cohort, 31 individuals (15.7%) reported regular oral contraceptive (OC) intake, while 166 individuals (84.3%) did not. In the OC cohort, 71.0% (22/31) were already taking OC regularly at the time of study enrolment, while in the no-OC cohort, 94.6% (157/166) were also part of the no-OC cohort at study enrolment.

The detailed characteristics of the second cross-sectional phase cohort are described in *Supplementary Table 2*, *Figure 1,* and *Supplementary Figure 1*.

Supplementary Figures and Supplementary Tables

Supplementary Table 1: Characterisation of the study population for overall usage of OC among HCWs in September 2023 (second cross-sectional phase)

The relative numbers in relation to the number of subjects of the study population are given in brackets following the absolute number. Age, BMI, and household size are given as medians with interquartile ranges in parentheses.

Where absolute figures are given, the corresponding relative figures are given in brackets. These always refer to the number of individuals in the respective categorical column. The exception is the relative numbers in relation to the column category, which refer to the total study population.

BMI: Body Mass Index [kg/m^2]

OC: oral contraceptives

	total	OC	no OC	p	p adj.
number of subjects	197 (100.0%)	31 (21.7%)	166 (78.3%)		
age (IQR) [years]	41 (32-45)	34 (30-41)	42 (33-46)	0.0052	0.11
BMI (IQR) [kg/m^2]	23.7 (21.3-26.3)	24.1 (21.6-21.6)	23.4 (21.2-26.0)	0.37	1.00
household size (IQR)	2 (1-4)	2 (1-4)	2 (1-4)	0.26	1.00
smoking					
smoking	23 (11.7%)	2 (6.5%)	21 (12.7%)	0.54	1.00
non-smoking	174 (88.3%)	29 (93.6%)	145 (87.4%)		
profession					
nursing	59 (30.0%)	7 (22.6%)	52 (31.3%)	0.066	0.72
physicians	20 (10.2%)	6 (19.4%)	14 (7.0%)		
other activity with regular patient contact	50 (25.4%)	4 (12.9%)	46 (27.7%)		
other activity without regular patient contact	68 (34.5%)	14 (45.2%)	54 (32.5%)		
frequency of patient contact					
never	47 (23.9%)	11 (35.5%)	36 (23.5%)	0.43	1.00
rare	24 (12.2%)	4 (12.9%)	20 (12.0%)		
regularly	27 (13.7%)	2 (6.5%)	25 (15.1%)		
often	11 (5.6%)	2 (6.5%)	9 (5.4%)		
very often	88 (44.7%)	12 (38.7%)	76 (45.8%)		
congenital or acquired immunodeficiency					
immunodeficiency	´1 (0.5%)	0 (0.0%)	1 (0.6%)	1.00	1.00
no immunodeficiency	196 (99.5%)	31 (100.0%)	165 (99.4%)		
long-term medication					
analgesics (e.g. ibuprofen, metamizole/ novaminesulfone)	9 (4.5%)	1 (3.2%)	8 (4.8%)	0.56	1.00
systemic immunosuppressive or modulating drugs (e.g. cortisone, antibody therapies)	2 (1.0%)	0 (0.0%)	2 (1.2%)		
inhalative or topical glucocorticoids (e.g. budesonide)	7 (3.6%)	2 (6.5%)	5 (3.0%)		
other	54 (27.4%)	6 (19.4%)	48 (28.9%)		

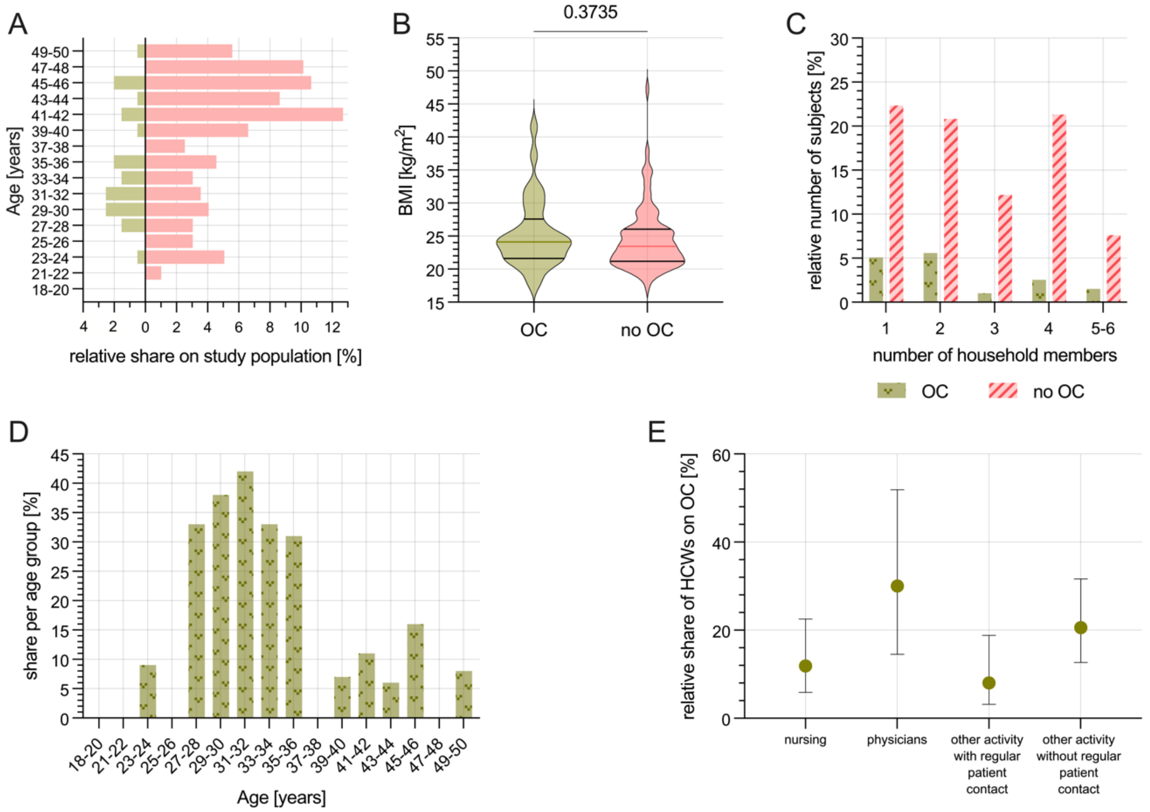

Supplementary Figure 1: Characterisation of the study population the second cross-section phase in September 2023 stratified by OC (individuals with OC use: n=31 (21.7%), individuals without OC use: n=166 (78.3%))

1A) Age structure stratified by OC
1B) BMI stratified by OC
1C) Relative share of HCWs stratified by number of household members and OC
1D) Relative share of HCWs with OC stratified by age group
1E) Relative share of subjects with OC stratified by profession
In the case of whiskers in the figures, these represent the respective 95% confidence interval.
BMI: Body Mass Index [kg/m^2]
OC: oral contraceptives

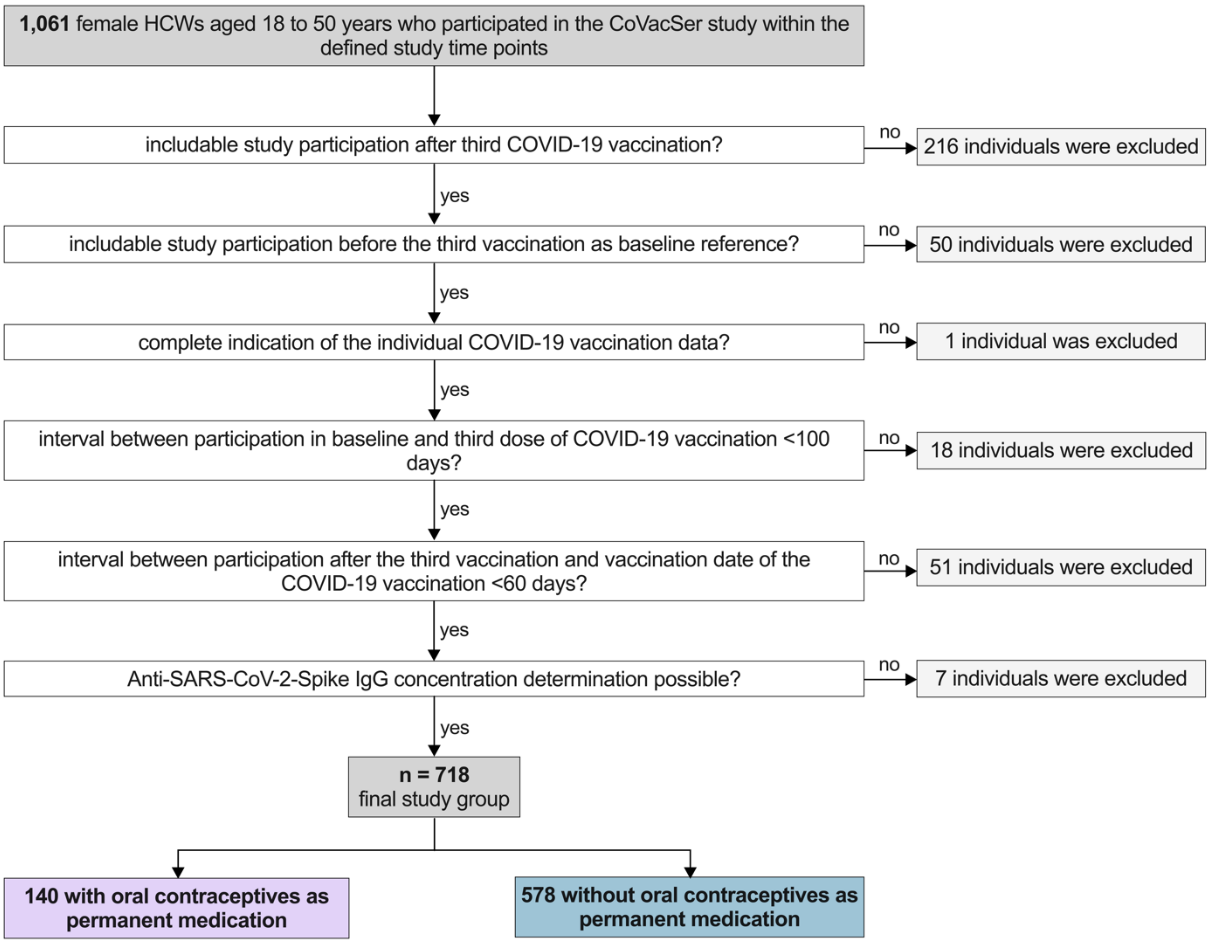

Supplementary Figure 2: Subject inclusion for the analysis related to the third COVID-19 vaccination

Supplementary Table 2: Characterisation of the study population of the analysis related to the third COVID-19 vaccination

The relative numbers in relation to the number of subjects of the study population are given in brackets following the absolute number. Age, BMI, household size, the interval of the pre-vaccination participation to third dose administration as well as the interval of the post-vaccination participation to third dose administration are given as medians with interquartile ranges in parentheses.

Where absolute figures are given, the corresponding relative figures are given in brackets. These always refer to the number of individuals in the respective categorical column. The exception is the relative numbers in relation to the column category, which refer to the total study population.

BMI: Body Mass Index [kg/m^2]

BAU/ml: Binding Antibody Units per millilitre

OC: oral contraceptives

	total	OC	no OC	p	p adj.
number of subjects	718 (100.0%)	140 (19.5%)	578 (80.5%)		
age (IQR) [years]	33 (27-41)	29 (26-34)	35 (28-42)	<0.0001	0.027
subjects aged <30 years	255 (35.5%)	72 (51.4%)	183 (31.7%)	<0.0001	0.027
BMI (IQR) [kg/m^2]	23.1 (20.9-26.4)	23.0 (20.8-26.2)	23.2 (20.9-26.5)	0.84	1.00
household size (IQR)	2 (2-4)	2 (1-3)	2 (2-4)	0.0005	0.0081
interval pre-vaccination participation to third dose administration (IQR) [days]	28 (16-41)	28 (16-41)	28 (16-41)	0.86	1.00
interval post-vaccination participation to third dose administration (IQR) [days]	18 (15-24)	19 (15-23)	18 (15-24)	0.46	1.00
SARS-CoV-2 infection history					
no SARS-CoV-2 infection	693 (96.5%)	134 (95.7%)	559 (96.7%)	0.61	1.00
one SARS-CoV-2 infection	25 (3.5%)	6 (4.3%)	19 (3.3%)		
first dose of COVID-19 vaccine					
BNT162b2mRNA	697 (97.1%)	135 (95.7%)	562 (97.2%)	0.87	1.00
mRNA-1273	9 (1.3%)	2 (1.4%)	7 (1.2%)		
ChAdOx1-S	12 (1.7%)	3 (2.1%)	9 (1.6%)		
second dose of COVID-19 vaccine					
BNT162b2mRNA	704 (98.1%)	136 (97.1%)	568 (98.1%)	0.43	1.00
mRNA-1273	11 (1.5%)	4 (2.9%)	7 (1.2%)		
ChAdOx1-S	2 (0.3%)	0 (0.0%)	2 (0.3%)		
Ad26.COV2-S	1 (0.1%)	0 (0.0%)	1 (0.2%)		
third dose of COVID-19 vaccine					
BNT162b2mRNA	586 (81.8%)	128 (91.4%)	458 (79.2%)	0.0006	0.0081
mRNA-1273	132 (18.2%)	12 (8.6%)	120 (20.8%)		
smoking					
smoking	83 (11.6%)	16 (11.4%)	67 (11.2%)	1.00	1.00
non-smoking	635 (88.4%)	124 (88.6%)	511 (88.4%)		
profession					
nursing	266 (37.0%)	49 (35.0%)	210 (36.3%)	0.59	1.00
physicians	121 (16.9%)	29 (20.7%)	92 (15.9%)		
other activity with regular patient contact	153 (21.3%)	28 (20.0%)	121 (20.9%)		
other activity without regular patient contact	191 (26.6%)	34 (24.3%)	155 (26.8%)		
Anti-SARS-CoV-2-Spike IgG					
pre vaccination [BAU/ml]	171 (92-271)	219 (111-336)	162 (88-261)	0.12	1.00
post vaccination [BAU/ml]	2,018 (1,314-3,145)	2,140 (1,356-3,261)	2,010 (1,306-3,078)	0.67	1.00
3-month follow-up [BAU/ml] (n=422)	832 (470-1,388)	869 (562-1,437)	792 (447-1,385)	0.30	1.00
6-month follow-up [BAU/ml] (n=198)	421 (265-954)	618 (280-1,432)	419 (254-903)	0.20	1.00

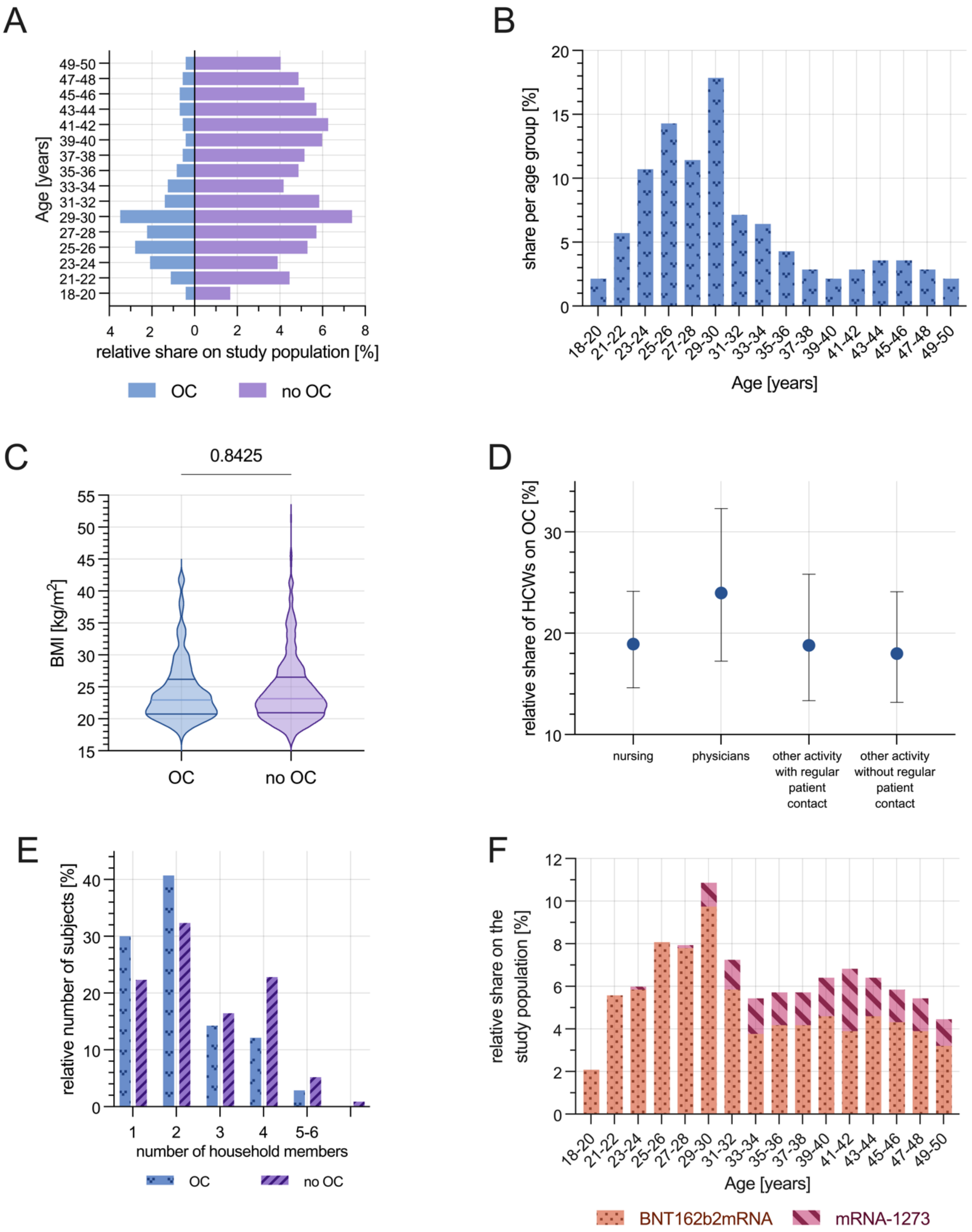

Supplementary Figure 3: Characterisation of the study population of the analysis related to the third COVID-19 vaccination (individuals with OC use: n=140 (19.5%), individuals without OC use: n=578 (80.5%))

3A) Age structure stratified by OC intake
3B) Relative share of HCWs with OC intake stratified by age group
3C) BMI stratified by OC intake
3D) Relative share of HCWs per age group with OC intake
3E) Relative share of HCWs stratified by number of household members and OC intake
3F) Relative share of HCWs stratified by third dose COVID-19 vaccine and age category

In the case of whiskers in the figures, these represent the respective 95% confidence interval.
BMI: Body Mass Index [kg/m^2]
OC: oral contraceptives

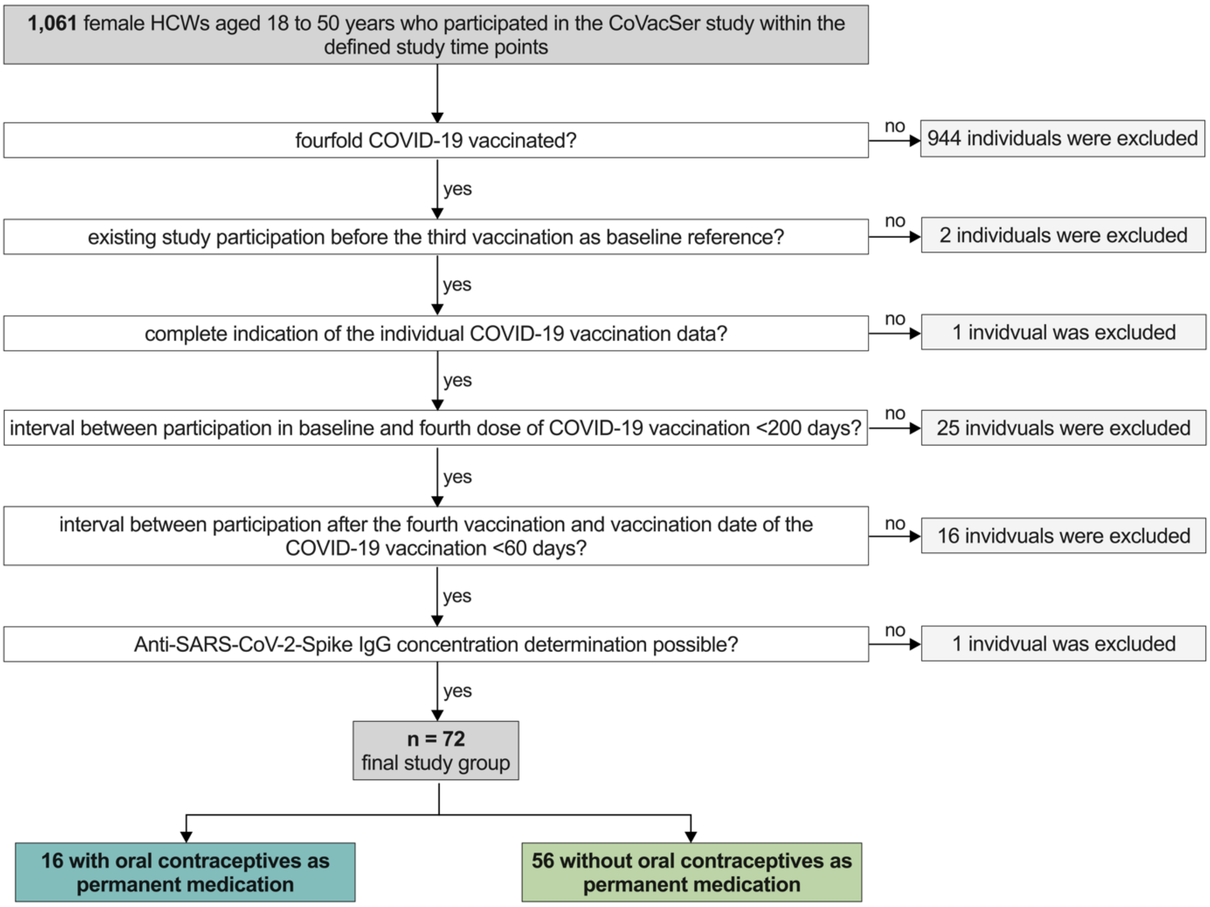

Supplementary Figure 4: Subject inclusion for the analysis related to the fourth COVID-19 vaccination

Supplementary Table 3: Characterisation of the study population of the analysis related to the fourth COVID-19 vaccination

The relative numbers in relation to the number of subjects of the study population are given in brackets following the absolute number. Age, BMI, household size, the interval of the pre-vaccination participation to third dose administration as well as the interval of the post-vaccination participation to third dose administration are given as medians with interquartile ranges in parentheses.

BAU/ml: Binding Antibody Units per Millilitre

BMI: Body Mass Index [kg/m^2]

OC: oral contraceptives

	**total**	**OC**	**no OC**	**p**	**p adj.**
number of subjects	72 (100.0%)	16 (21.2%)	56 (78.8%)		
age (IQR) [years]	40 (32-46)	34 (29-43)	42 (33-46)	0.054	1.00
BMI (IQR) [kg/m^2]	22.8 (20.8-26.4)	24.4 (22.2-29.7)	22.4 (20.7-25.9)	0.093	1.00
household size (IQR)	2 (1-4)	2 (1-2)	3 (2-4)	0.018	0.84
interval pre-vaccination participation to third dose administration (IQR) [days]	98 (30-135)	110 (74-145)	74 (23-131)	0.16	1.00
interval post-vaccination participation to third dose administration (IQR) [days]	21 (18-28)	23 (17-32)	21 (18-27)	0.60	1.00
SARS-CoV-2 infection history					
no SARS-CoV-2 infection	36 (50.0%)	10 (62.5%)	26 (46.4%)	0.41	1.00
one SARS-CoV-2 infection	32 (44.4%)	5 (31. %)	27 (48.2%)		
two SARS-CoV-2 infections	4 (5.6%)	1 (6.7%)	3 (5.4%)		
first dose of COVID-19 vaccine					
BNT162b2mRNA	72 (100.0%)	16 (100.0%)	56 (100.0%)		
second dose of COVID-19 vaccine					
BNT162b2mRNA	72 (100.0%)	16 (100.0%)	56 (100.0%)		
third dose of COVID-19 vaccine					
BNT162b2mRNA	70 (97.2%)	16 (100.0%)	54 (96.4%)	0.44	1.00
mRNA-1273	2 (2.8%)	0 (0.0%)	2 (3.6%)		
fourth dose of COVID-19 vaccine					
BNT162b2mRNA	51 (70.8%)	14 (87.5%)	37 (66.1%)	0.37	1.00
mRNA-1273	4 (5.6%)	0 (0.0%)	4 (7.1%)		
BNT162b2mRNA Original/Omicron BA.1	1 (1.4%)	0 (0.0%)	1 (1.8%)		
BNT162b2mRNA Original/Omicron BA.4-5	16 (22.2%)	2 (12.5%)	14 (25.0%)		
smoking					
smoking	11 (15.3%)	2 (12.5%)	9 (16.1%)	1.00	1.00
non-smoking	61 (84.7%)	14 (87.5%)	47 (83.9%)		
profession					
nursing	27 (37.5%)	6 (37.5%)	21 (37.5%)	0.66	1.00
physicians	20 (27.8%)	6 (37.5%)	14 (25.0%)		
other activity with regular patient contact	10 (13.9%)	1 (6.25%)	9 (16.1%)		
other activity without regular patient contact	15 (20.8%)	3 (18.75%)	12 (21.4%)		
Anti-SARS-CoV-2-Spike IgG level					
pre vaccination [BAU/ml]	934 (493-1,752)	996 (502-1,621)	934 (493-1,775)	0.84	1.00
post vaccination [BAU/ml]	2,947 (1,793-4,065)	3,806 (2,608-4,708)	2,619 (1,634-3,979)	0.12	1.00
3-month follow-up [BAU/ml]	1,653 (1,216-3,108)	1,751 (1,415-3,625)	1,654 (1,108-2,953)	0.65	1.00
6-month follow-up [BAU/ml]	1,341 (872-2,186)	2,504 (1,422-9,780)	1,186 (721-1,864)	0.092	1.00

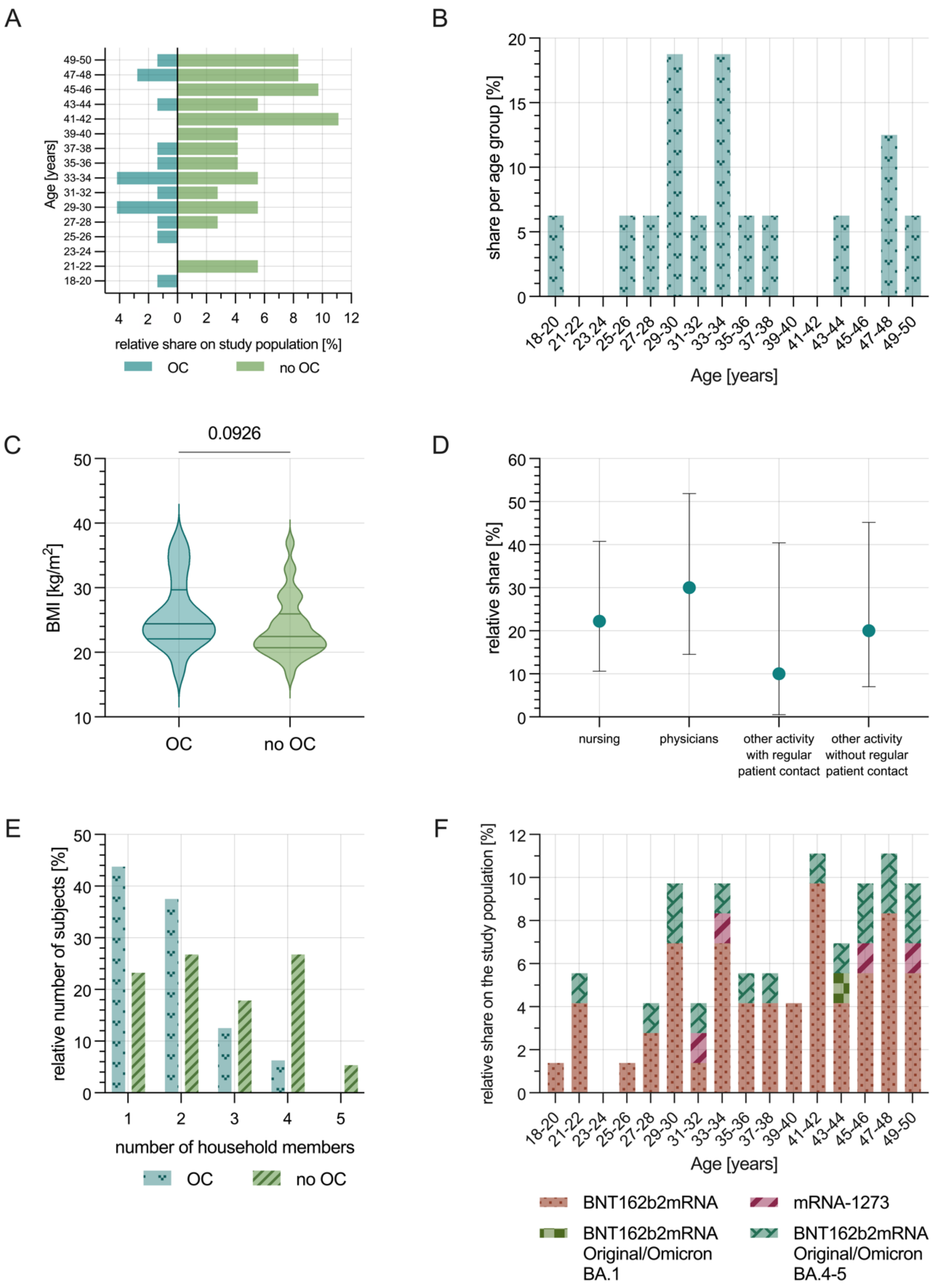

Supplementary Figure 5: Characterisation of the study population of the analysis related to the fourth COVID-19 vaccination (individuals with OC use: n=16 (21.2%), individuals without OC use: n=56 (78.8%))

5A) Age structure stratified by OC intake
5B) Relative share of HCWs with OC intake stratified by age group
5C) BMI stratified by OC intake
5D) Relative share of HCWs per age group with OC intake
5E) Relative share of HCWs stratified by number of household members and OC intake
5F) Relative share of HCWs stratified by fourth dose COVID-19 vaccine and age category

In the case of whiskers in the figures, these represent the respective 95% confidence interval.
BMI: Body Mass Index [kg/m^2]
OC: oral contraceptives

Supplementary Table 4: Characterisation of the T-SPOT®.COVID sub-cohort

The relative numbers in relation to the number of subjects of the study population are given in brackets following the absolute number. Age, BMI, household size, the interval of the pre-vaccination participation to third dose administration as well as the interval of the post-vaccination participation to third dose administration are given as medians with interquartile ranges in parentheses.

BMI: Body Mass Index [kg/m^2]

BAU: Binding Antibody Units per millilitre

OC: oral contraceptives

	total	OC	no OC	p	p adj.
number of subjects	201 (100.0%)	34 (16.9%)	179 (89.1%)		
age (IQR) [years]	36 (30-42)	31 (26-39)	36 (30-42)	0.0063	0.26
BMI (IQR) [kg/m^2]	23.0 (20.7-26.8)	23.4 (20.6-25.9)	23.0 (20.8-27.1)	0.94	1.00
household size (IQR)	2 (2-4)	2 (1-3)	2 (2-4)	0.091	1.00
days since last COVID-19 immunising event (IQR) [days]	91 (77-103)	92 (84-102)	91 (75-103)	0.84	1.00
SARS-CoV-2 infection history					
no SARS-CoV-2 infection	147 (73.1%)	27 (79.4%)	120 (71.9%)	0.41	1.00
one SARS-CoV-2 infection	54 (26.9%)	7 (20.6%)	47 (28.1%)		
first dose of COVID-19 vaccine					
BNT162b2mRNA	196 (97.5%)	33 (97.1%)	163 (81.1%)	1.00	1.00
ChAdOx1-S	5 (2.5%)	1 (2.9%)	4 (2.0%)		
second dose of COVID-19 vaccine					
BNT162b2mRNA	197 (98.0%)	33 (97.1%)	164 (98.2%)	0.37	1.00
mRNA-1273	4 (1.0%)	1 (2.9%)	1 (0.6%)		
ChAdOx1-S	2 (1.0%)	0 (0.0%)	2 (1.2%)		
third dose of COVID-19 vaccine					
BNT162b2mRNA	149 (74.1%)	25 (73.5%)	124 (74.3%)	1.00	1.00
mRNA-1273	52 (25.9%)	9 (26.5%)	43 (25.7%)		
smoking					
smoking	17 (8.5%)	2 (5.9%)	17 (9.0%)	0.74	1.00
non-smoking	184 (91.5%)	38 (94.1%)	162 (91.0%)		
profession					
nursing	47 (23.8%)	6 (17.6%)	41 (24.6%)	0.19	1.00
physicians	22 (10.9%)	7 (20.6%)	15 (9.0%)		
other activity with regular patient contact	48 (23.9%)	6 (17.6%)	42 (25.2%)		
other activity without regular patient contact	84 (41.8%)	15 (44.1%)	69 (41.3%)		
Anti-SARS-CoV-2-Spike IgG level					
Anti-SARS-CoV-2-Spike IgG [BAU/ml]	951 (461-2,016)	833 (478-1,300)	999 (448-2,168)	0.31	1.00
T-SPOT®.COVID assay					
Anti-SARS-CoV-2-Spike SFU	104 (50-228)	122 (55-230)	104 (48-228)	0.69	1.00
Anti-SARS-CoV-2-Nucleocapsid SFU	4 (0-24)	6 (0-19)	4 (0-24)	0.48	1.00

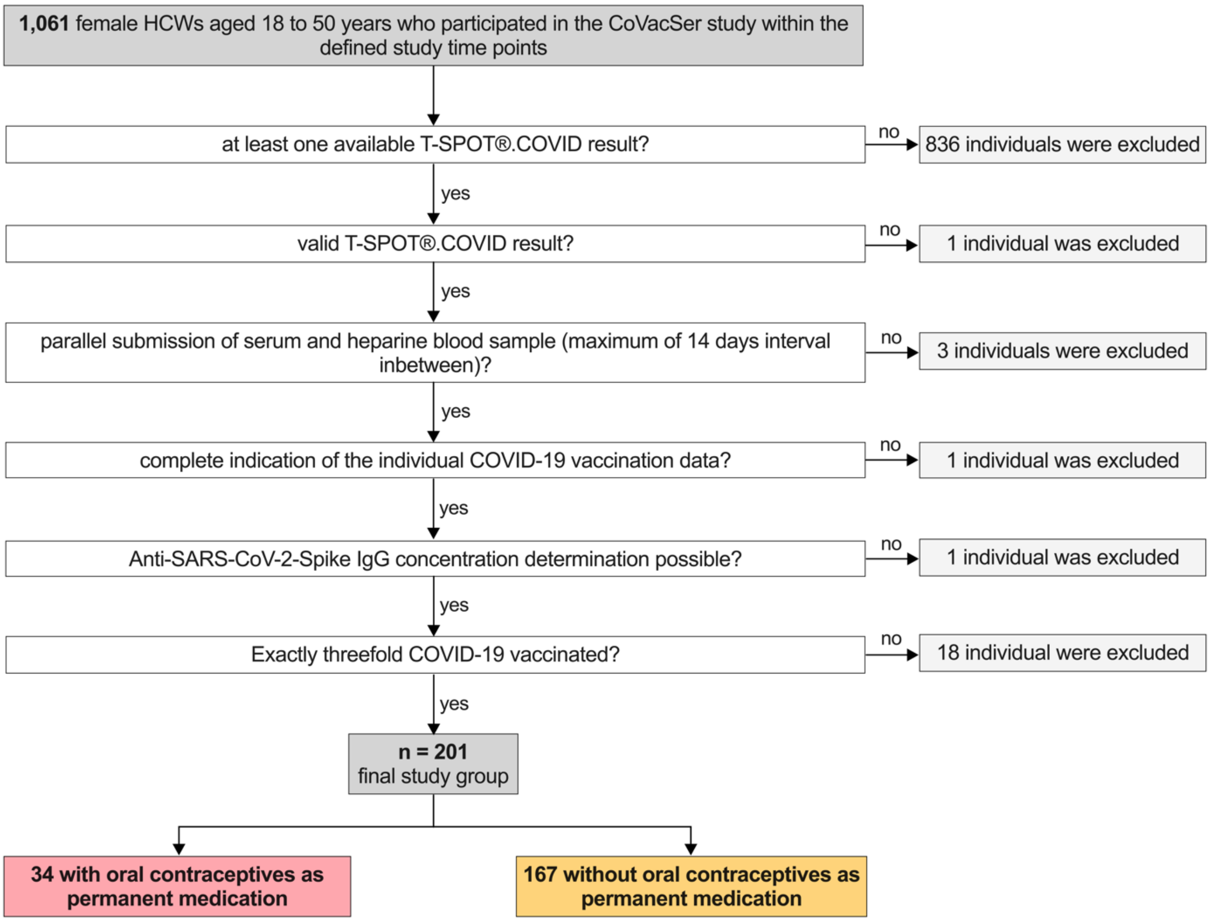

Supplementary Figure 6: Subject inclusion for the analysis related to the T-SPOT®.COVID cohort

In the case of whiskers in the figures, these represent the respective 95% confidence interval.

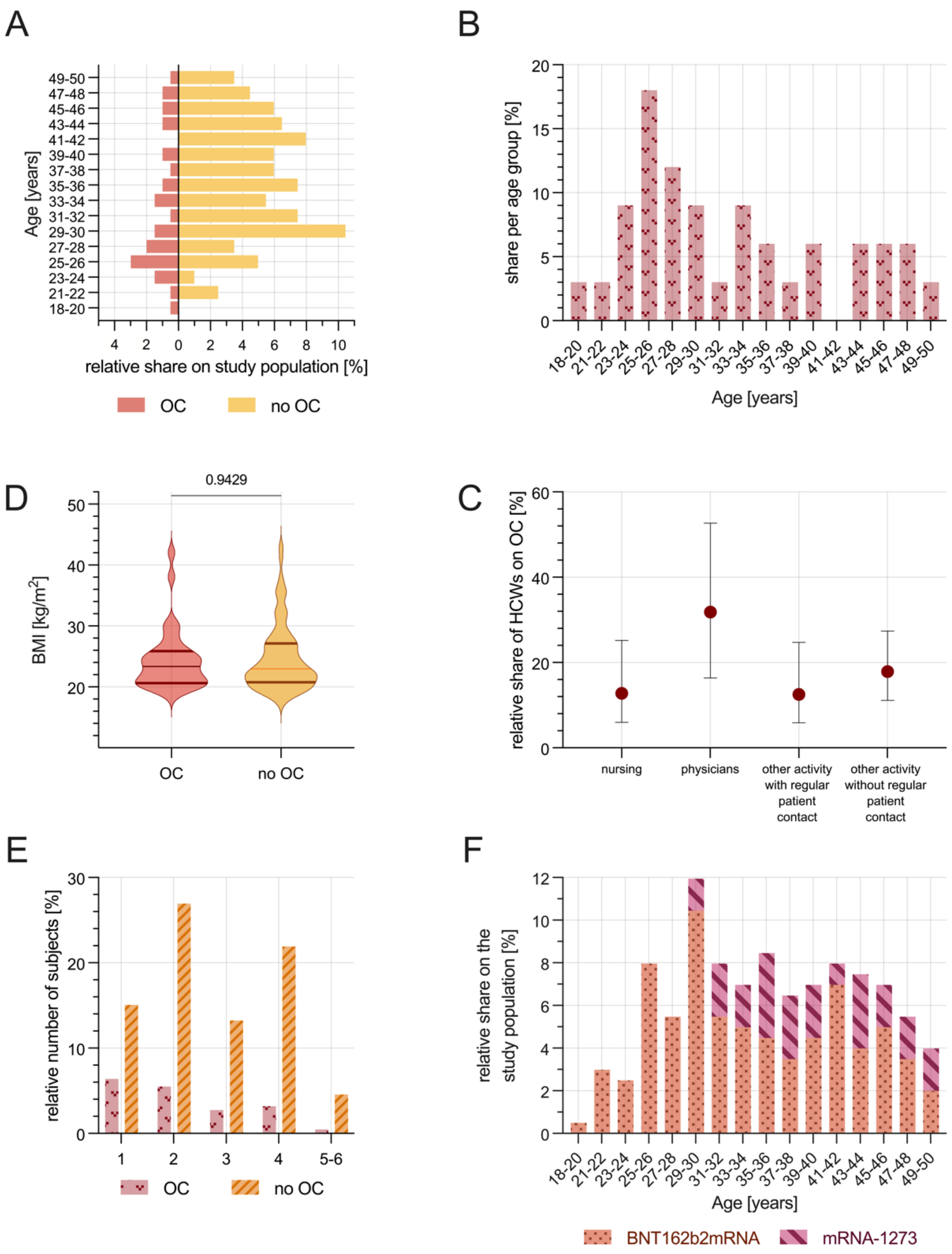

Supplementary Figure 7: Characterisation of the study population of the analysis related to the T-SPOT®.COVID cohort (individuals with OC use: n=34 (16.9%), individuals without OC use: n=179 (89.1%))

7A) Age structure stratified by OC intake
7B) Relative share of HCWs with OC intake stratified by age group
7C) BMI stratified by OC intake
7D) Relative share of HCWs per age group with OC intake
7E) Relative share of HCWs stratified by number of household members and OC intake
7F) Relative share of HCWs stratified by third dose COVID-19 vaccine and age category

In the case of whiskers in the figures, these represent the respective 95% confidence interval.
BMI: Body Mass Index [kg/m^2]
OC: oral contraceptives
