# Supplementary material for: Oral contraceptive usage among healthcare workers and its impact on COVID-19 booster vaccination immunogenicity
Source: NPJ Vaccines. 2026 Jul 2;11:134. doi: 10.1038/s41541-026-01510-z (PMC13328437; doi:10.1038/s41541-026-01510-z)
Supplement: Supplementary file 2 — Supplementary statistical analysis [file 41541_2026_1510_MOESM2_ESM.html]

Statistical Analysis: Usage of Oral Contraceptives among Healthcare Workers and the Impact on B- and T-cellular Immunogenicity after COVID-19 Booster Vaccinations


Code 

- Show All Code
- Hide All Code

# Statistical Analysis: Usage of Oral Contraceptives among Healthcare Workers and the Impact on B- and T-cellular Immunogenicity after COVID-19 Booster Vaccinations

#### Alexander Gabel

#### 01-03-2024

```
options(scipen = 999)
library(dplyr)
library(ggplot2)
library(lubridate)
library(glmnet)
library(emmeans)
# required:
# ggstance
# broom.mixed
# jtools
# effects
# sjPlot
# rstatix

super_dir <- "~/CoVacSer/OC_impact_paper/"
plot.dir <- file.path(super_dir, "plots")
data.dir <- file.path(super_dir, "data")

if(!file.exists(data.dir)){
  dir.create(data.dir, recursive = T)
}

for(plt_format in c("png", "pdf", "svg")){

  if(!file.exists(file.path(plot.dir, plt_format))){
    dir.create(file.path(plot.dir, plt_format), recursive = T)
  }
}
```

# Impact IgG after third vaccination

```
df_input_3rdVacc <- readxl::read_excel(file.path(data.dir,"AG_16_Datenauszug_nachDritterImpfung_20240121_V2.xlsx"))

df_ana <- df_input_3rdVacc %>% dplyr::select(MasterID, Infektion, BMI, Alter, Haushalt, Dauermed_4, Rauchen, Beruf, IgG_pre, IgG_post, `impfstoff_#3`) %>%
                             dplyr::mutate(Dauermed_4 = factor(Dauermed_4),
                                           Beruf = factor(Beruf),
                                           IgG_pre = log10(IgG_pre),
                                           IgG_post = log10(IgG_post),
                                           Infektion = factor(Infektion),
                                           MasterID = as.character(MasterID),
                                           `impfstoff_#3` = factor(`impfstoff_#3`)) %>%
                       dplyr::rename(`infection_history` = Infektion,
                                     `oc_intake` = Dauermed_4,
                                     profession = Beruf,
                                     smoking = Rauchen,
                                     household_size = Haushalt,
                                     age = Alter,
                                     bmi = BMI,
                                     third_vaccine = `impfstoff_#3`) %>% 
                       tidyr::pivot_longer(cols = c(IgG_pre, IgG_post), 
                                           names_to = "time", values_to = "IgG") %>%
                       mutate(time = factor(time, levels = c("IgG_pre", "IgG_post")))
```

# Multiple regression model

```
mixed_model <- lme4::lmer(IgG ~ bmi + age + household_size + smoking + profession + 
                           time + infection_history + oc_intake + time:infection_history  + 
                           time:oc_intake + (1|MasterID),
                           REML = T, data = df_ana)
```

## Check assumptions for multiple regression analysis

### Test for Autocorrelation (Durbin-Watson)

```
model_residuals <- as.numeric(residuals(mixed_model))
dw_stat <- car::durbinWatsonTest(model_residuals)

# Calculate P-value based on permutation
n_obs <- length(model_residuals)
n_samplings <- 1000
mu <- fitted.values(mixed_model)
X_model <- model.matrix(IgG ~  ., data = df_ana %>% dplyr::select(-MasterID))

sampled_data <-  matrix(sample(model_residuals, n_obs * n_samplings, replace = TRUE), n_obs, n_samplings) + matrix(mu, n_obs, n_samplings)

E <- residuals(nlme::gls(sampled_data ~ X_model - 1, weights = nlme::varPower()))
dw_sampled <- apply(E, 2, car::durbinWatsonTest)

p_value_dw <- (sum(dw_sampled > dw_stat))/n_samplings
p_value_dw <- 2 * (min(p_value_dw, 1 - p_value_dw))

hist(dw_sampled, breaks = 50, freq = T, main = "Histogram sampled DW statistics", xlab = "Sampled DW values", col = "white")
abline(v = dw_stat, lwd = 3, col = "red")
```

**Durbin-Watson-Test for autocorrelation:**

**Test statistic:** ***d =*** 2.32
Indicates positive autocorrelation (probably pre to post IgG
levels).

**P-value:**  ***p =*** 0

## Normality of residuals

```
x_vals <- seq(min(model_residuals), max(model_residuals), length.out = 1000)

hist(model_residuals, xlab = "Residuals", prob = T, col = "white", main = expression('Density of residuals'), breaks = 30)
lines(x = x_vals, y = dnorm(x = x_vals, mean = mean(model_residuals), sd = sd(model_residuals)), lty = 1, lwd = 2)
```

```
png(filename = file.path(plot.dir, "png/histogram_residuals.png"), units="px", width=1600, height=1600, res=300)
hist(model_residuals, xlab = "Residuals", prob = T, col = "white", main = expression('Density of residuals'), breaks = 30)
lines(x = x_vals, y = dnorm(x = x_vals, mean = mean(model_residuals), sd = sd(model_residuals)), lty = 1, lwd = 2)
dev.off()
```

## Variance of residuals

```
data.frame(fit = mu, residuals = model_residuals) %>% 
  ggplot2::ggplot(aes(x = fit, y = residuals)) + 
  ggplot2::geom_point(alpha = 0.75, pch = 20, size = 2) + 
  ggplot2::xlab("Fitted values based on linear model") + 
  ggplot2::ylab("Residuals from linear model") + 
  ggplot2::theme_bw() +
  ggplot2::theme(axis.text = ggplot2::element_text(size = 14, colour = "black"),
                 axis.title = ggplot2::element_text(face = "bold", size = 12),
                 legend.title = ggplot2::element_blank(),
                 legend.text = ggplot2::element_text(size = 16),
                 legend.position = "bottom")
```

```
sapply(c("png", "pdf", "svg"), function(i){
  ggplot2::ggsave(plot = last_plot(), filename = file.path(plot.dir, i, paste0("Generalized_linear_model_full_model_variance.",i)), device = i,  width = 10, height = 8, dpi = 300)
})
```

## Simplest model:

```
gls_simple <- nlme::gls(IgG ~ time * oc_intake,
                      data = df_ana,
                      weights = nlme::varPower())
```

### Coefficients of the model:

```
sum_gls <- summary(gls_simple)
glm_p_values <- cbind(sum_gls$tTable, p.adj = p.adjust(sum_gls$tTable[,4], method = "BY"))

anova_gls <- as.data.frame(anova(gls_simple))
anova_gls <- cbind(anova_gls, p.adj = p.adjust(anova_gls$`p-value`, method = "BY"))
anova_gls <- anova_gls %>% mutate(p_plot = dplyr::case_when(p.adj < 1e-4 ~ "< 0.0001",
                                             p.adj > 1e-4 &  p.adj < 1e-3  ~"< 0.001",
                                             p.adj > 1e-3 &  p.adj < 1e-2  ~ "< 0.01",
                                             p.adj > 1e-2 &  p.adj < 0.05  ~ "< 0.05",
                                             TRUE ~ format(round(p.adj, 2))
                                             ))

y_hat_igg <- predict(gls_simple, interval = "prediction") 
rsquare_cv <- cor(df_ana$IgG, y_hat_igg)^2

anova_gls %>% 
   DT::datatable(extensions = 'Buttons', options = list(
    dom = 'Blfrtip',
    buttons = c('copy', 'csv', 'excel', 'pdf'),
    lengthMenu = list(c(10,30, 50, -1), 
                      c('10', '30', '50', 'All')),
    paging = F))
```

## Posthoc tests: Pairwise comparisons within discrete factors

- compare subgroups within discrete factors
- estimate marginal means for each factor and compare the
  subgroups

```
entities <- c("time", "oc_intake", "time:oc_intake")
marg_list <- list()
for(i in seq_along(entities)){
  marginal <- emmeans(gls_simple, eval(parse( text = paste0("~ ", entities[i]))), rg.limit = 160000)
  marg_list[[i]] <- as.data.frame(pairs(marginal))
}

names(marg_list) <- entities

post_hoc_comp <- do.call("rbind", marg_list)

post_hoc_comp$contrast <- gsub(pattern = "\\(|\\)", replacement = "", x = post_hoc_comp$contrast)
post_hoc_comp <- cbind(post_hoc_comp, p.adj = p.adjust(post_hoc_comp$p.value, method = "BY"))

post_hoc_comp %>% 
   DT::datatable(extensions = 'Buttons', options = list(
    dom = 'Blfrtip',
    buttons = c('copy', 'csv', 'excel', 'pdf'),
    lengthMenu = list(c(10,30, 50, -1), 
                      c('10', '30', '50', 'All')),
    paging = F))
```

## Complexer models:

```
gls_full_add <- nlme::gls(IgG ~ bmi + age + household_size + smoking + profession + third_vaccine + infection_history + time + oc_intake,
                      data = df_ana,
                      weights = nlme::varPower())

gls_full_int1 <- nlme::gls(IgG ~ bmi + age + household_size + smoking + profession + third_vaccine + infection_history + time * oc_intake,
                      data = df_ana,
                      weights = nlme::varPower())

gls_full_int2 <- nlme::gls(IgG ~ bmi + age + household_size + smoking + profession + third_vaccine + infection_history * time  + oc_intake,
                      data = df_ana,
                      weights = nlme::varPower())

gls_full_int3 <- nlme::gls(IgG ~ bmi + age + household_size + smoking + profession + time + third_vaccine + infection_history + oc_intake + time:infection_history + time:oc_intake,
                      data = df_ana,
                      weights = nlme::varPower())

gls_full_int4 <- nlme::gls(IgG ~ bmi + age + household_size + smoking + profession + third_vaccine + time * infection_history * oc_intake,
                      data = df_ana,
                      weights = nlme::varPower())

mixed_model <- nlme::lme(IgG ~ bmi + age + household_size + smoking + profession + third_vaccine +  
                           time + infection_history + oc_intake + time:infection_history  + 
                           time:oc_intake, random = ~1|MasterID, data = df_ana)

model_comparisons <- cbind(AIC(gls_simple, gls_full_add, gls_full_int1, 
                               gls_full_int2, gls_full_int3, gls_full_int4, mixed_model), 
                           BIC(gls_simple, gls_full_add, gls_full_int1, 
                               gls_full_int2, gls_full_int3, gls_full_int4, mixed_model)[, 2, drop = F])
rownames(model_comparisons) <- c("simple model", "additive model", "add model + time:OC", 
                                 "add model + time:infection_history", "add model + time:OC + time:infection_history", 
                                 "add model + time:OC + time:infection_history + time:infection_history:OC", 
                                 "mixed model+ time:OC + time:infection_history")
model_comparisons %>% 
   DT::datatable(extensions = 'Buttons', options = list(
    dom = 'Blfrtip',
    buttons = c('copy', 'csv', 'excel', 'pdf'),
    lengthMenu = list(c(10,30, 50, -1), 
                      c('10', '30', '50', 'All')),
    paging = F))
```

## Mixed Effects Model (time:OC + time:infection\_history):

```
sum_gls <- summary(mixed_model)
glm_p_values <- cbind(sum_gls$tTable, p.adj = p.adjust(sum_gls$tTable[,4], method = "BY"))

anova_gls <- as.data.frame(anova(mixed_model))
anova_gls <- cbind(anova_gls, p.adj = p.adjust(anova_gls$`p-value`, method = "BY"))
anova_gls <- anova_gls %>% mutate(p_plot = dplyr::case_when(p.adj < 1e-4 ~ "< 0.0001",
                                             p.adj > 1e-4 &  p.adj < 1e-3  ~"< 0.001",
                                             p.adj > 1e-3 &  p.adj < 1e-2  ~ "< 0.01",
                                             p.adj > 1e-2 &  p.adj < 0.05  ~ "< 0.05",
                                             TRUE ~ format(round(p.adj, 2))
                                             ))

y_hat_igg <- predict(mixed_model, interval = "prediction") 
rsquare_cv <- cor(df_ana$IgG, y_hat_igg)^2

anova_gls %>% 
   DT::datatable(extensions = 'Buttons', options = list(
    dom = 'Blfrtip',
    buttons = c('copy', 'csv', 'excel', 'pdf'),
    lengthMenu = list(c(10,30, 50, -1), 
                      c('10', '30', '50', 'All')),
    paging = F))
```

**R^2 = 0.87**

## Posthoc tests: Pairwise comparisons within discrete factors

```
entities <- c("profession", "smoking", "third_vaccine", "infection_history", "time", "oc_intake", "time:oc_intake", "time:infection_history")
marg_list <- list()
for(i in seq_along(entities)){
  marginal <- emmeans(mixed_model, eval(parse( text = paste0("~ ", entities[i]))), rg.limit = 160000)
  marg_list[[i]] <- as.data.frame(pairs(marginal))
}

names(marg_list) <- entities

post_hoc_comp <- do.call("rbind", marg_list)

post_hoc_comp$contrast <- gsub(pattern = "\\(|\\)", replacement = "", x = post_hoc_comp$contrast)
post_hoc_comp <- cbind(post_hoc_comp, p.adj = p.adjust(post_hoc_comp$p.value, method = "BY"))

profession_labels <- paste0("Profession: ", apply(combn(x = c("Nursing", "Physicians", "others pat. contact", 
                                                              "others no pat. contact"), m = 2),  2, function(col_el){
  paste0(col_el, collapse = " vs. ")
}))

single_labels <- c("smoker vs. non-smoker", "third_vaccine", "NoInfection - Infection", "IgG_pre - IgG_post", "noOC vs. OC")

contrast_labels <- c(profession_labels, single_labels, post_hoc_comp$contrast[-c(1:11)])
post_hoc_comp <- cbind(comparison = contrast_labels, post_hoc_comp)

post_hoc_comp  %>% 
   DT::datatable(extensions = 'Buttons', options = list(
    dom = 'Blfrtip',
    buttons = c('copy', 'csv', 'excel', 'pdf'),
    lengthMenu = list(c(10,30, 50, -1), 
                      c('10', '30', '50', 'All')),
    paging = F))
```

## Coefficients pairwise comparisons (All)

```
post_hoc_comp <- post_hoc_comp[rev(seq_len(nrow(post_hoc_comp))),]
post_hoc_comp$comparison <- factor(post_hoc_comp$comparison, levels = post_hoc_comp$comparison) 

post_hoc_comp %>% ggplot2::ggplot() +
  ggplot2::geom_point(ggplot2::aes(y = comparison, x = estimate)) +
  ggplot2::geom_errorbar(aes(y = comparison, xmin=estimate-SE, xmax=estimate+SE), width=.2,
                         position=position_dodge(.9)) +
  ggplot2::theme_bw() +
  ggplot2::theme(axis.text = ggplot2::element_text(size = 12, colour = "black"),
                 axis.title = ggplot2::element_text(face = "bold", size = 12),
                 legend.title = ggplot2::element_blank(),
                 legend.text = ggplot2::element_text(size = 12),
                 legend.position = "bottom") +
  ggplot2::xlab("Effect on IgG (estimated coefficient difference)") + 
  ggplot2::ylab("Comparison")
```

```
sapply(c("png", "pdf", "svg"), function(i){
  ggplot2::ggsave(plot = last_plot(), filename = file.path(plot.dir, i, paste0("Generalized_linear_model_full_model_all_comparisons.",i)), device = i,  width = 10, height = 12, dpi = 300)
})
```

## including 3month follow up

```
df_ana <- df_input_3rdVacc %>% dplyr::select(MasterID, Infektion, BMI, Alter, Haushalt, 
                                     Dauermed_4, Rauchen, Beruf, IgG_pre, IgG_post, IgG_3month, `impfstoff_#3`) %>%
                             dplyr::mutate(Dauermed_4 = factor(Dauermed_4),
                                           Beruf = factor(Beruf),
                                           IgG_pre = log10(IgG_pre),
                                           IgG_post = log10(IgG_post),
                                           Infektion = if_else(Infektion == 0, true = 0, false = 1),
                                           Infektion = factor(Infektion),
                                           MasterID = as.character(MasterID),
                                           `impfstoff_#3` = factor(`impfstoff_#3`)) %>%
                       dplyr::rename(`infection_history` = Infektion,
                                     `oc_intake` = Dauermed_4,
                                     profession = Beruf,
                                     smoking = Rauchen,
                                     household_size = Haushalt,
                                     age = Alter,
                                     bmi = BMI,
                                     third_vaccine = `impfstoff_#3`) %>% 
                       tidyr::pivot_longer(cols = c(IgG_pre, IgG_post, IgG_3month), 
                                           names_to = "time", values_to = "IgG") %>%
                       mutate(time = factor(time, levels = c("IgG_pre", "IgG_post", "IgG_3month"))) %>%
                       na.omit()

mixed_model_3month <- nlme::lme(IgG ~ bmi + age + household_size + smoking + profession + third_vaccine +
                           time + infection_history + oc_intake + time:infection_history  + 
                           time:oc_intake, random = ~1|MasterID, data = df_ana)
```

## Model (time:OC + time:infection\_history):

```
sum_gls <- summary(mixed_model_3month)
glm_p_values <- cbind(sum_gls$tTable, p.adj = p.adjust(sum_gls$tTable[,4], method = "BY"))

anova_gls <- as.data.frame(anova(mixed_model_3month))
anova_gls <- cbind(anova_gls, p.adj = p.adjust(anova_gls$`p-value`, method = "BY"))
anova_gls <- anova_gls %>% mutate(p_plot = dplyr::case_when(p.adj < 1e-4 ~ "< 0.0001",
                                             p.adj > 1e-4 &  p.adj < 1e-3  ~"< 0.001",
                                             p.adj > 1e-3 &  p.adj < 1e-2  ~ "< 0.01",
                                             p.adj > 1e-2 &  p.adj < 0.05  ~ "< 0.05",
                                             TRUE ~ format(round(p.adj, 2))
                                             ))

y_hat_igg <- predict(mixed_model_3month, interval = "prediction") 
rsquare_cv <- cor(df_ana$IgG, y_hat_igg)^2

anova_gls  %>% 
   DT::datatable(extensions = 'Buttons', options = list(
    dom = 'Blfrtip',
    buttons = c('copy', 'csv', 'excel', 'pdf'),
    lengthMenu = list(c(10,30, 50, -1), 
                      c('10', '30', '50', 'All')),
    paging = F))
```

**R^2 = 0.47**

## Posthoc tests: Pairwise comparisons within discrete factors

```
entities <- c("profession", "smoking", "third_vaccine" , "infection_history", "time", "oc_intake", "time:oc_intake", "time:infection_history")
marg_list <- list()
for(i in seq_along(entities)){
  marginal <- emmeans(mixed_model_3month, eval(parse( text = paste0("~ ", entities[i]))), rg.limit = 160000)
  marg_list[[i]] <- as.data.frame(pairs(marginal))
}

names(marg_list) <- entities

post_hoc_comp <- do.call("rbind", marg_list)

post_hoc_comp$contrast <- gsub(pattern = "\\(|\\)", replacement = "", x = post_hoc_comp$contrast)
post_hoc_comp <- cbind(post_hoc_comp, p.adj = p.adjust(post_hoc_comp$p.value, method = "BY"))

profession_labels <- paste0("Profession: ", apply(combn(x = c("Nursing", "Physicians", "others pat. contact", 
                                                              "others no pat. contact"), m = 2),  2, function(col_el){
  paste0(col_el, collapse = " vs. ")
}))

single_labels <- c("smoker vs. non-smoker", "BioNTech - Moderna", "NoInfection - Infection", 
                   "IgG_pre - IgG_post", " IgG_pre - IgG_3month", "IgG_post - IgG_3month", "noOC vs. OC")

contrast_labels <- c(profession_labels, single_labels, post_hoc_comp$contrast[-c(1:13)])
post_hoc_comp <- cbind(comparison = contrast_labels, post_hoc_comp)

post_hoc_comp  %>% 
   DT::datatable(extensions = 'Buttons', options = list(
    dom = 'Blfrtip',
    buttons = c('copy', 'csv', 'excel', 'pdf'),
    lengthMenu = list(c(10,30, 50, -1), 
                      c('10', '30', '50', 'All')),
    paging = F))
```

## Coefficients pairwise comparisons (All)

```
post_hoc_comp <- post_hoc_comp[rev(seq_len(nrow(post_hoc_comp))),]
post_hoc_comp$comparison <- factor(post_hoc_comp$comparison, levels = post_hoc_comp$comparison) 

post_hoc_comp %>% ggplot2::ggplot() +
  ggplot2::geom_point(ggplot2::aes(y = comparison, x = estimate)) +
  ggplot2::geom_errorbar(aes(y = comparison, xmin=estimate-SE, xmax=estimate+SE), width=.2,
                         position=position_dodge(.9)) +
  ggplot2::theme_bw() +
  ggplot2::theme(axis.text = ggplot2::element_text(size = 12, colour = "black"),
                 axis.title = ggplot2::element_text(face = "bold", size = 12),
                 legend.title = ggplot2::element_blank(),
                 legend.text = ggplot2::element_text(size = 12),
                 legend.position = "bottom") +
  ggplot2::xlab("Effect on IgG (estimated coefficient difference)") + 
  ggplot2::ylab("Comparison")
```

```
sapply(c("png", "pdf", "svg"), function(i){
  ggplot2::ggsave(plot = last_plot(), filename = file.path(plot.dir, i, paste0("Generalized_linear_model_full_model_all_comparisons.",i)), device = i,  width = 10, height = 12, dpi = 300)
})
```

## including 3month follow up

```
df_ana <- df_input_3rdVacc %>% dplyr::select(MasterID, Infektion, BMI, Alter, Haushalt, 
                                     Dauermed_4, Rauchen, Beruf, IgG_pre, IgG_post, IgG_3month, IgG_6month4, `impfstoff_#3`) %>%
                             dplyr::mutate(Dauermed_4 = factor(Dauermed_4),
                                           Beruf = factor(Beruf),
                                           IgG_pre = log10(IgG_pre),
                                           IgG_post = log10(IgG_post),
                                           Infektion = if_else(Infektion == 0, true = 0, false = 1),
                                           Infektion = factor(Infektion),
                                           MasterID = as.character(MasterID),
                                           `impfstoff_#3` = factor(`impfstoff_#3`)) %>%
                       dplyr::rename(`infection_history` = Infektion,
                                     `oc_intake` = Dauermed_4,
                                     profession = Beruf,
                                     smoking = Rauchen,
                                     household_size = Haushalt,
                                     age = Alter,
                                     bmi = BMI,
                                     third_vaccine = `impfstoff_#3`) %>% 
                       tidyr::pivot_longer(cols = c(IgG_pre, IgG_post, IgG_3month), 
                                           names_to = "time", values_to = "IgG") %>%
                       mutate(time = factor(time, levels = c("IgG_pre", "IgG_post", "IgG_3month", "IgG_6month4"))) %>%
                       na.omit()

mixed_model_6month <- nlme::lme(IgG ~ bmi + age + household_size + smoking + profession + third_vaccine +
                           time + infection_history + oc_intake + time:infection_history  + 
                           time:oc_intake, random = ~1|MasterID, data = df_ana)
```

## Mixed Model (time:OC + time:infection\_history):

```
sum_gls <- summary(mixed_model_6month)
glm_p_values <- cbind(sum_gls$tTable, p.adj = p.adjust(sum_gls$tTable[,4], method = "BY"))

anova_gls <- as.data.frame(anova(mixed_model_6month))
anova_gls <- cbind(anova_gls, p.adj = p.adjust(anova_gls$`p-value`, method = "BY"))
anova_gls <- anova_gls %>% mutate(p_plot = dplyr::case_when(p.adj < 1e-4 ~ "< 0.0001",
                                             p.adj > 1e-4 &  p.adj < 1e-3  ~"< 0.001",
                                             p.adj > 1e-3 &  p.adj < 1e-2  ~ "< 0.01",
                                             p.adj > 1e-2 &  p.adj < 0.05  ~ "< 0.05",
                                             TRUE ~ format(round(p.adj, 2))
                                             ))

y_hat_igg <- predict(mixed_model_6month, interval = "prediction") 
rsquare_cv <- cor(df_ana$IgG, y_hat_igg)^2

anova_gls  %>% 
   DT::datatable(extensions = 'Buttons', options = list(
    dom = 'Blfrtip',
    buttons = c('copy', 'csv', 'excel', 'pdf'),
    lengthMenu = list(c(10,30, 50, -1), 
                      c('10', '30', '50', 'All')),
    paging = F))
```

**R^2 = 0.44**

## Posthoc tests: Pairwise comparisons within discrete factors

```
entities <- c("profession", "smoking", "third_vaccine", "infection_history", "time", "oc_intake", "time:oc_intake", "time:infection_history")
marg_list <- list()
for(i in seq_along(entities)){
  marginal <- emmeans(mixed_model_6month, eval(parse( text = paste0("~ ", entities[i]))), rg.limit = 160000)
  marg_list[[i]] <- as.data.frame(pairs(marginal))
}

names(marg_list) <- entities

post_hoc_comp <- do.call("rbind", marg_list)

post_hoc_comp$contrast <- gsub(pattern = "\\(|\\)", replacement = "", x = post_hoc_comp$contrast)
post_hoc_comp <- cbind(post_hoc_comp, p.adj = p.adjust(post_hoc_comp$p.value, method = "BY"))

profession_labels <- paste0("Profession: ", apply(combn(x = c("Nursing", "Physicians", "others pat. contact", 
                                                              "others no pat. contact"), m = 2),  2, function(col_el){
  paste0(col_el, collapse = " vs. ")
}))

single_labels <- c("smoker vs. non-smoker", "BioNTech - Moderna", "NoInfection - Infection", 
                   "IgG_pre - IgG_post", "IgG_pre - IgG_3month", "IgG_post - IgG_3month", "noOC vs. OC")

contrast_labels <- c(profession_labels, single_labels, post_hoc_comp$contrast[-c(1:13)])
post_hoc_comp <- cbind(comparison = contrast_labels, post_hoc_comp)

post_hoc_comp  %>% 
   DT::datatable(extensions = 'Buttons', options = list(
    dom = 'Blfrtip',
    buttons = c('copy', 'csv', 'excel', 'pdf'),
    lengthMenu = list(c(10,30, 50, -1), 
                      c('10', '30', '50', 'All')),
    paging = F))
```

## Coefficients pairwise comparisons (All)

```
post_hoc_comp <- post_hoc_comp[rev(seq_len(nrow(post_hoc_comp))),]
post_hoc_comp$comparison <- factor(post_hoc_comp$comparison, levels = post_hoc_comp$comparison) 

post_hoc_comp %>% ggplot2::ggplot() +
  ggplot2::geom_point(ggplot2::aes(y = comparison, x = estimate)) +
  ggplot2::geom_errorbar(aes(y = comparison, xmin=estimate-SE, xmax=estimate+SE), width=.2,
                         position=position_dodge(.9)) +
  ggplot2::theme_bw() +
  ggplot2::theme(axis.text = ggplot2::element_text(size = 12, colour = "black"),
                 axis.title = ggplot2::element_text(face = "bold", size = 12),
                 legend.title = ggplot2::element_blank(),
                 legend.text = ggplot2::element_text(size = 12),
                 legend.position = "bottom") +
  ggplot2::xlab("Effect on IgG (estimated coefficient difference)") + 
  ggplot2::ylab("Comparison")
```

```
sapply(c("png", "pdf", "svg"), function(i){
  ggplot2::ggsave(plot = last_plot(), filename = file.path(plot.dir, i, paste0("Generalized_linear_model_full_model_all_comparisons.",i)), device = i,  width = 10, height = 12, dpi = 300)
})
```

# Impact IgG after fourth vaccination

```
df_input <- readxl::read_excel(file.path(data.dir,"AG_16_Datenauszug_nachVIERTERImpfung_20240121_V1.xlsx"))

df_ana <- df_input %>% dplyr::select(MasterID, Infektion, BMI, Alter, Haushalt, Dauermed_42, Rauchen, Beruf, IgG_pre, IgG_post, impfstoff_4) %>%
                       dplyr::filter(impfstoff_4 %in% 1:2) %>%
                             dplyr::mutate(Dauermed_42 = factor(Dauermed_42),
                                           Beruf = factor(Beruf),
                                           IgG_pre = log10(IgG_pre),
                                           IgG_post = log10(IgG_post),
                                           Infektion = if_else(Infektion == 0, true = 0, false = 1),
                                           Infektion = factor(Infektion), 
                                           impfstoff_4 = factor(impfstoff_4)) %>%
                       dplyr::rename(`infection_history` = Infektion,
                                     `oc_intake` = Dauermed_42,
                                     profession = Beruf,
                                     smoking = Rauchen,
                                     household_size = Haushalt,
                                     age = Alter,
                                     bmi = BMI,
                                     fourth_vaccine = impfstoff_4) %>% 
                       tidyr::pivot_longer(cols = c(IgG_pre, IgG_post), 
                                           names_to = "time", values_to = "IgG") %>%
                       mutate(time = factor(time, levels = c("IgG_pre", "IgG_post")))
```

# Multiple regression model

```
gls_full_add <- nlme::gls(IgG ~ bmi + age + household_size + smoking + profession + fourth_vaccine + infection_history + time + oc_intake,
                      data = df_ana,
                      weights = nlme::varPower())

gls_full_int1 <- nlme::gls(IgG ~ bmi + age + household_size + smoking + profession + fourth_vaccine + infection_history + time * oc_intake,
                      data = df_ana,
                      weights = nlme::varPower())

gls_full_int2 <- nlme::gls(IgG ~ bmi + age + household_size + smoking + profession + fourth_vaccine + infection_history * time  + oc_intake,
                      data = df_ana,
                      weights = nlme::varPower())

gls_full_int3 <- nlme::gls(IgG ~ bmi + age + household_size + smoking + profession + fourth_vaccine + time + infection_history + oc_intake + time:infection_history + time:oc_intake,
                      data = df_ana,
                      weights = nlme::varPower())

gls_full_int4 <- nlme::gls(IgG ~ bmi + age + household_size + smoking + profession + fourth_vaccine + time * infection_history * oc_intake,
                      data = df_ana,
                      weights = nlme::varPower())

mixed_model_4th_vacc <- nlme::lme(IgG ~ bmi + age + household_size + smoking + profession + fourth_vaccine +
                                        time + infection_history + oc_intake + time:infection_history  + 
                                        time:oc_intake, random = ~1|MasterID, data = df_ana)

model_comparisons <- cbind(AIC(gls_full_add, gls_full_int1, gls_full_int2, gls_full_int3, gls_full_int4, mixed_model_4th_vacc), 
                   BIC(gls_full_add, gls_full_int1, gls_full_int2, gls_full_int3, gls_full_int4, mixed_model_4th_vacc)[, 2, drop = F])
rownames(model_comparisons) <- c("additive model", "add model + time:OC", 
                                 "add model + time:infection_history", "add model + time:OC + time:infection_history", 
                                 "add model + time:OC + time:infection_history + time:infection_history:OC",
                                 "mixed model + time:OC + time:infection_history")
model_comparisons  %>% 
   DT::datatable(extensions = 'Buttons', options = list(
    dom = 'Blfrtip',
    buttons = c('copy', 'csv', 'excel', 'pdf'),
    lengthMenu = list(c(10,30, 50, -1), 
                      c('10', '30', '50', 'All')),
    paging = F))
```

## Check assumptions for multiple regression analysis

### Test for Autocorrelation (Durbin-Watson)

```
model_residuals <- as.numeric(residuals(mixed_model_4th_vacc))
dw_stat <- car::durbinWatsonTest(model_residuals)

# Calculate P-value based on permutation
n_obs <- length(model_residuals)
n_samplings <- 1000
mu <- fitted.values(mixed_model_4th_vacc)
X_model <- model.matrix(IgG ~  ., data = df_ana %>% dplyr::select(-MasterID))

sampled_data <-  matrix(sample(model_residuals, n_obs * n_samplings, replace = TRUE), n_obs, n_samplings) + matrix(mu, n_obs, n_samplings)

E <- residuals(nlme::gls(sampled_data ~ X_model - 1, weights = nlme::varPower()))
dw_sampled <- apply(E, 2, car::durbinWatsonTest)

p_value_dw <- (sum(dw_sampled > dw_stat))/n_samplings
p_value_dw <- 2 * (min(p_value_dw, 1 - p_value_dw))

hist(dw_sampled, breaks = 50, freq = T, main = "Histogram sampled DW statistics", xlab = "Sampled DW values", col = "white")
abline(v = dw_stat, lwd = 3, col = "red")
```

**Durbin-Watson-Test for autocorrelation (no
autocorrelation):**

**Test statistic:** ***d =***2.39

**P-value:**  ***p =*** 0

## Normality of residuals

```
x_vals <- seq(min(model_residuals), max(model_residuals), length.out = 1000)

hist(model_residuals, xlab = "Residuals", prob = T, col = "white", main = expression('Density of residuals'), breaks = 30)
lines(x = x_vals, y = dnorm(x = x_vals, mean = mean(model_residuals), sd = sd(model_residuals)), lty = 1, lwd = 2)
```

```
png(filename = file.path(plot.dir, "png/histogram_residuals.png"), units="px", width=1600, height=1600, res=300)
hist(model_residuals, xlab = "Residuals", prob = T, col = "white", main = expression('Density of residuals'), breaks = 30)
lines(x = x_vals, y = dnorm(x = x_vals, mean = mean(model_residuals), sd = sd(model_residuals)), lty = 1, lwd = 2)
dev.off()
```

## Variance of residuals

```
data.frame(fit = mu, residuals = model_residuals) %>% 
  ggplot2::ggplot(aes(x = fit, y = residuals)) + 
  ggplot2::geom_point(alpha = 0.75, pch = 20, size = 2) + 
  ggplot2::xlab("Fitted values based on linear model") + 
  ggplot2::ylab("Residuals from linear model") + 
  ggplot2::theme_bw() +
  ggplot2::theme(axis.text = ggplot2::element_text(size = 14, colour = "black"),
                 axis.title = ggplot2::element_text(face = "bold", size = 12),
                 legend.title = ggplot2::element_blank(),
                 legend.text = ggplot2::element_text(size = 16),
                 legend.position = "bottom")
```

```
sapply(c("png", "pdf", "svg"), function(i){
  ggplot2::ggsave(plot = last_plot(), filename = file.path(plot.dir, i, paste0("Generalized_linear_model_full_model_variance.",i)), device = i,  width = 10, height = 8, dpi = 300)
})
```

## Coefficients of the model:

```
sum_gls <- summary(mixed_model_4th_vacc)
glm_p_values <- cbind(sum_gls$tTable, p.adj = p.adjust(sum_gls$tTable[,4], method = "BY"))

anova_gls <- as.data.frame(anova(mixed_model_4th_vacc))
anova_gls <- cbind(anova_gls, p.adj = p.adjust(anova_gls$`p-value`, method = "BY"))
anova_gls <- anova_gls %>% mutate(p_plot = dplyr::case_when(p.adj < 1e-4 ~ "< 0.0001",
                                             p.adj > 1e-4 &  p.adj < 1e-3  ~"< 0.001",
                                             p.adj > 1e-3 &  p.adj < 1e-2  ~ "< 0.01",
                                             p.adj > 1e-2 &  p.adj < 0.05  ~ "< 0.05",
                                             TRUE ~ format(round(p.adj, 2))
                                             ))

y_hat_igg <- predict(mixed_model_4th_vacc, interval = "prediction") 
rsquare_cv <- cor(df_ana$IgG, y_hat_igg)^2

anova_gls  %>% 
   DT::datatable(extensions = 'Buttons', options = list(
    dom = 'Blfrtip',
    buttons = c('copy', 'csv', 'excel', 'pdf'),
    lengthMenu = list(c(10,30, 50, -1), 
                      c('10', '30', '50', 'All')),
    paging = F))
```

**R^2 = 0.75**

## Posthoc tests: Pairwise comparisons within discrete factors

```
entities <- c("profession", "smoking", "fourth_vaccine", "infection_history", "time", "oc_intake", "time:oc_intake", "time:infection_history")
marg_list <- list()
for(i in seq_along(entities)){
  marginal <- emmeans(mixed_model_4th_vacc, eval(parse( text = paste0("~ ", entities[i]))), rg.limit = 160000)
  marg_list[[i]] <- as.data.frame(pairs(marginal))
}

names(marg_list) <- entities

post_hoc_comp <- do.call("rbind", marg_list)

post_hoc_comp$contrast <- gsub(pattern = "\\(|\\)", replacement = "", x = post_hoc_comp$contrast)
post_hoc_comp <- cbind(post_hoc_comp, p.adj = p.adjust(post_hoc_comp$p.value, method = "BY"))

profession_labels <- paste0("Profession: ", apply(combn(x = c("Nursing", "Physicians", "others pat. contact", 
                                                              "others no pat. contact"), m = 2),  2, function(col_el){
  paste0(col_el, collapse = " vs. ")
}))

single_labels <- c("smoker vs. non-smoker", "BioNTech - Moderna", "NoInfection - Infection", "IgG_pre - IgG_post", "noOC vs. OC")

contrast_labels <- c(profession_labels, single_labels, post_hoc_comp$contrast[-c(1:11)])
post_hoc_comp <- cbind(comparison = contrast_labels, post_hoc_comp)

post_hoc_comp  %>% 
   DT::datatable(extensions = 'Buttons', options = list(
    dom = 'Blfrtip',
    buttons = c('copy', 'csv', 'excel', 'pdf'),
    lengthMenu = list(c(10,30, 50, -1), 
                      c('10', '30', '50', 'All')),
    paging = F))
```

## Coefficients pairwise comparisons (All)

```
post_hoc_comp <- post_hoc_comp[rev(seq_len(nrow(post_hoc_comp))),]
post_hoc_comp$comparison <- factor(post_hoc_comp$comparison, levels = post_hoc_comp$comparison) 

post_hoc_comp %>% ggplot2::ggplot() +
  ggplot2::geom_point(ggplot2::aes(y = comparison, x = estimate)) +
  ggplot2::geom_errorbar(aes(y = comparison, xmin=estimate-SE, xmax=estimate+SE), width=.2,
                         position=position_dodge(.9)) +
  ggplot2::theme_bw() +
  ggplot2::theme(axis.text = ggplot2::element_text(size = 12, colour = "black"),
                 axis.title = ggplot2::element_text(face = "bold", size = 12),
                 legend.title = ggplot2::element_blank(),
                 legend.text = ggplot2::element_text(size = 12),
                 legend.position = "bottom") +
  ggplot2::xlab("Effect on IgG (estimated coefficient difference)") + 
  ggplot2::ylab("Comparison")
```

```
sapply(c("png", "pdf", "svg"), function(i){
  ggplot2::ggsave(plot = last_plot(), filename = file.path(plot.dir, i, paste0("Generalized_linear_model_full_model_all_comparisons.",i)), device = i,  width = 10, height = 12, dpi = 300)
})
```

# T cell response - Anti-SARS-CoV-2-Spike

```
df_input <- readxl::read_excel(file.path(data.dir,"AG_16_Datenauszug_T-SPOT_20240121_V2.xlsx"))

df_ana <- df_input %>% dplyr::select(MasterID, infektion, BMI, Alter, haushalt, OC_dauermedikation___4, 
                                     rauchen, beruf,
                                     Days_since_last_event, impfstoff3, SFU_PanelA,  SFU_PanelB) %>%
                             dplyr::mutate(OC_dauermedikation___4 = factor(OC_dauermedikation___4),
                                           beruf = factor(beruf),
                                           infektion = factor(infektion),
                                           impfstoff3 = factor(impfstoff3),
                                           rauchen = factor(rauchen)) %>%
                       dplyr::rename(`infection_history` = infektion,
                                     `3rd dose vaccine` = impfstoff3,
                                     `oc_intake` = OC_dauermedikation___4,
                                     profession = beruf,
                                     smoking = rauchen,
                                     household_size = haushalt,
                                     age = Alter,
                                     bmi = BMI,
                                     AntiS = SFU_PanelA,
                                     AntiN = SFU_PanelB)
```

## NB Regression model (Negative-Binomial)

```
gls_full_nb <- MASS::glm.nb(AntiS ~ bmi + age + household_size + smoking + profession + Days_since_last_event + 
                                   `3rd dose vaccine` + infection_history + oc_intake,
                      data = df_ana)
```

## Coefficients of the model:

```
sum_gls <- summary(gls_full_nb)
glm_p_values <- cbind(sum_gls$tTable, p.adj = p.adjust(sum_gls$tTable[,4], method = "BY"))

anova_gls <- suppressWarnings(as.data.frame(anova(gls_full_nb)))
anova_gls <- cbind(anova_gls, p.adj = p.adjust(anova_gls$`Pr(>Chi)`, method = "BY"))
anova_gls <- anova_gls %>% mutate(p_plot = dplyr::case_when(p.adj < 1e-4 ~ "< 0.0001",
                                                            p.adj > 1e-4 &  p.adj < 1e-3  ~"< 0.001",
                                                            p.adj > 1e-3 &  p.adj < 1e-2  ~ "< 0.01",
                                                            p.adj > 1e-2 &  p.adj < 0.05  ~ "< 0.05",
                                                            TRUE ~ format(round(p.adj, 2))
                                                           )
                                    )

y_hat_igg <- predict(gls_full_nb, interval = "prediction") 
rsquare <- with(summary(gls_full_nb), 1 - deviance/null.deviance)

anova_gls  %>% 
   DT::datatable(extensions = 'Buttons', options = list(
    dom = 'Blfrtip',
    buttons = c('copy', 'csv', 'excel', 'pdf'),
    lengthMenu = list(c(10,30, 50, -1), 
                      c('10', '30', '50', 'All')),
    paging = F))
```

**R^2 = 0.19**

## Posthoc tests: Pairwise comparisons within discrete factors

- compare subgroups within discrete factors
- estimate marginal means for each factor and compare the
  subgroups

```
entities <- c("oc_intake", "smoking", "infection_history", "`3rd dose vaccine`", "profession")
marg_list <- list()
for(i in seq_along(entities)){
  marginal <- emmeans(gls_full_nb, eval(parse( text = paste0("~ ", entities[i]))), rg.limit = 160000)
  marg_list[[i]] <- as.data.frame(pairs(marginal))
}

names(marg_list) <- entities

post_hoc_comp <- do.call("rbind", marg_list)

post_hoc_comp$contrast <- gsub(pattern = "\\(|\\)", replacement = "", x = post_hoc_comp$contrast)
post_hoc_comp <- cbind(post_hoc_comp, p.adj = p.adjust(post_hoc_comp$p.value, method = "BY"))

contrast_labels <- c("OC intake (No vs. Yes)", "Smoking (Yes vs. No)")

infection_labels <- paste0("Infection history: ", apply(combn(x = 0:1, m = 2),  2, function(col_el){
  paste0(col_el, collapse = " vs. ")
}))

third_vaccine <- c("3rd dose vaccine: BioNTech - Moderna")

profession_labels <- paste0("Profession: ", apply(combn(x = c("Nursing", "Physicians", "others pat. contact", "others no pat. contact"), m = 2),  2, function(col_el){
  paste0(col_el, collapse = " vs. ")
}))

contrast_labels <- c(contrast_labels, infection_labels, third_vaccine, profession_labels)

post_hoc_comp <- cbind(comparison = contrast_labels, post_hoc_comp)

post_hoc_comp  %>% 
   DT::datatable(extensions = 'Buttons', options = list(
    dom = 'Blfrtip',
    buttons = c('copy', 'csv', 'excel', 'pdf'),
    lengthMenu = list(c(10,30, 50, -1), 
                      c('10', '30', '50', 'All')),
    paging = F))
```

## Coefficients pairwise comparisons (All)

```
post_hoc_comp <- post_hoc_comp[rev(seq_len(nrow(post_hoc_comp))),]
post_hoc_comp$comparison <- factor(post_hoc_comp$comparison, levels = post_hoc_comp$comparison) 

post_hoc_comp %>% ggplot2::ggplot() +
  ggplot2::geom_point(ggplot2::aes(y = comparison, x = estimate)) +
  ggplot2::geom_errorbar(aes(y = comparison, xmin=estimate-SE, xmax=estimate+SE), width=.2,
                         position=position_dodge(.9)) +
  ggplot2::theme_bw() +
  ggplot2::theme(axis.text = ggplot2::element_text(size = 12, colour = "black"),
                 axis.title = ggplot2::element_text(face = "bold", size = 12),
                 legend.title = ggplot2::element_blank(),
                 legend.text = ggplot2::element_text(size = 12),
                 legend.position = "bottom") +
  ggplot2::xlab("Effect on SUF (estimated coefficient difference)") + 
  ggplot2::ylab("Comparison")
```

```
sapply(c("png", "pdf", "svg"), function(i){
  ggplot2::ggsave(plot = last_plot(), filename = file.path(plot.dir, i, paste0("Generalized_linear_model_full_model_all_comparisons.",i)), device = i,  width = 10, height = 12, dpi = 300)
})
```

# T cell response - Anti-SARS-CoV-2-Nucleocapsid

# NB Regression model (Negative-Binomial)

```
gls_full_nb <- MASS::glm.nb(AntiN ~ bmi + age + household_size + smoking + profession + Days_since_last_event + `3rd dose vaccine` + infection_history + oc_intake,
                      data = df_ana)
```

## Coefficients of the model:

```
sum_gls <- summary(gls_full_nb)
glm_p_values <- cbind(sum_gls$tTable, p.adj = p.adjust(sum_gls$tTable[,4], method = "BY"))

anova_gls <- suppressWarnings(as.data.frame(anova(gls_full_nb)))
anova_gls <- cbind(anova_gls, p.adj = p.adjust(anova_gls$`Pr(>Chi)`, method = "BY"))
anova_gls <- anova_gls %>% mutate(p_plot = dplyr::case_when(p.adj < 1e-4 ~ "< 0.0001",
                                                            p.adj > 1e-4 &  p.adj < 1e-3  ~"< 0.001",
                                                            p.adj > 1e-3 &  p.adj < 1e-2  ~ "< 0.01",
                                                            p.adj > 1e-2 &  p.adj < 0.05  ~ "< 0.05",
                                                            TRUE ~ format(round(p.adj, 2))
                                                           )
                                    )

y_hat_igg <- predict(gls_full_nb, interval = "prediction") 
rsquare <- with(summary(gls_full_nb), 1 - deviance/null.deviance)

anova_gls  %>% 
   DT::datatable(extensions = 'Buttons', options = list(
    dom = 'Blfrtip',
    buttons = c('copy', 'csv', 'excel', 'pdf'),
    lengthMenu = list(c(10,30, 50, -1), 
                      c('10', '30', '50', 'All')),
    paging = F))
```

**R^2 = 0.27**

## Posthoc tests: Pairwise comparisons within discrete factors

- compare subgroups within discrete factors
- estimate marginal means for each factor and compare the
  subgroups

```
entities <- c("oc_intake", "smoking", "infection_history", "`3rd dose vaccine`", "profession")
marg_list <- list()
for(i in seq_along(entities)){
  marginal <- emmeans(gls_full_nb, eval(parse( text = paste0("~ ", entities[i]))), rg.limit = 160000)
  marg_list[[i]] <- as.data.frame(pairs(marginal))
}

names(marg_list) <- entities

post_hoc_comp <- do.call("rbind", marg_list)

post_hoc_comp$contrast <- gsub(pattern = "\\(|\\)", replacement = "", x = post_hoc_comp$contrast)
post_hoc_comp <- cbind(post_hoc_comp, p.adj = p.adjust(post_hoc_comp$p.value, method = "BY"))

contrast_labels <- c("OC intake (No vs. Yes)", "Smoking (Yes vs. No)")

infection_labels <- paste0("Infection history: ", apply(combn(x = 0:1, m = 2),  2, function(col_el){
  paste0(col_el, collapse = " vs. ")
}))

third_vaccine <- c("3rd dose vaccine: BioNTech - Moderna")

profession_labels <- paste0("Profession: ", apply(combn(x = c("Nursing", "Physicians", "others pat. contact", "others no pat. contact"), m = 2),  2, function(col_el){
  paste0(col_el, collapse = " vs. ")
}))

contrast_labels <- c(contrast_labels, infection_labels, third_vaccine, profession_labels)

post_hoc_comp <- cbind(comparison = contrast_labels, post_hoc_comp)

post_hoc_comp   %>% 
   DT::datatable(extensions = 'Buttons', options = list(
    dom = 'Blfrtip',
    buttons = c('copy', 'csv', 'excel', 'pdf'),
    lengthMenu = list(c(10,30, 50, -1), 
                      c('10', '30', '50', 'All')),
    paging = F))
```

## Coefficients pairwise comparisons (All)

```
post_hoc_comp <- post_hoc_comp[rev(seq_len(nrow(post_hoc_comp))),]
post_hoc_comp$comparison <- factor(post_hoc_comp$comparison, levels = post_hoc_comp$comparison) 

post_hoc_comp %>% ggplot2::ggplot() +
  ggplot2::geom_point(ggplot2::aes(y = comparison, x = estimate)) +
  ggplot2::geom_errorbar(aes(y = comparison, xmin=estimate-SE, xmax=estimate+SE), width=.2,
                         position=position_dodge(.9)) +
  ggplot2::theme_bw() +
  ggplot2::theme(axis.text = ggplot2::element_text(size = 12, colour = "black"),
                 axis.title = ggplot2::element_text(face = "bold", size = 12),
                 legend.title = ggplot2::element_blank(),
                 legend.text = ggplot2::element_text(size = 12),
                 legend.position = "bottom") +
  ggplot2::xlab("Effect on SUF (estimated coefficient difference)") + 
  ggplot2::ylab("Comparison")
```

```
sapply(c("png", "pdf", "svg"), function(i){
  ggplot2::ggsave(plot = last_plot(), filename = file.path(plot.dir, i, paste0("Generalized_linear_model_full_model_all_comparisons.",i)), device = i,  width = 10, height = 12, dpi = 300)
})
```
